# Reduction of primary graft dysfunction using cytokine adsorption during organ preservation and after lung transplantation

**DOI:** 10.1038/s41467-022-31811-5

**Published:** 2022-07-26

**Authors:** Haider Ghaidan, Martin Stenlo, Anna Niroomand, Margareta Mittendorfer, Gabriel Hirdman, Nika Gvazava, Dag Edström, Iran A. N. Silva, Ellen Broberg, Oskar Hallgren, Franziska Olm, Darcy E. Wagner, Leif Pierre, Snejana Hyllén, Sandra Lindstedt

**Affiliations:** 1 Department of Cardiothoracic Surgery and Transplantation, Skåne University Hospital, Lund, Sweden; 2 Wallenberg Center for Molecular Medicine, Lund University, Lund, Sweden; 3 Department of Clinical Sciences, Lund University, Lund, Sweden; 4 Lund Stem Cell Center, Lund University, Lund, Sweden; 5 Department of Cardiothoracic Anaesthesia and Intensive Care, Skåne University Hospital, Lund, Sweden; 6 Rutgers Robert University, New Brunswick, NJ USA; 7 Department of Experimental Medical Sciences, Lung Bioengineering and Regeneration, Lund University, Lund, Sweden

**Keywords:** Organ transplantation, Experimental models of disease, Translational research

## Abstract

Despite improvements, lung transplantation remains hampered by both a scarcity of donor organs and by mortality following primary graft dysfunction (PGD). Since acute respiratory distress syndrome (ARDS) limits donor lungs utilization, we investigated cytokine adsorption as a means of treating ARDS donor lungs. We induced mild to moderate ARDS using lipopolysaccharide in 16 donor pigs. Lungs were then treated with or without cytokine adsorption during ex vivo lung perfusion (EVLP) and/or post-transplantation using extracorporeal hemoperfusion. The treatment significantly decreased cytokine levels during EVLP and decreased levels of immune cells post-transplantation. Histology demonstrated fewer signs of lung injury across both treatment periods and the incidence of PGD was significantly reduced among treated animals. Overall, cytokine adsorption was able to restore lung function and reduce PGD in lung transplantation. We suggest this treatment will increase the availability of donor lungs and increase the tolerability of donor lungs in the recipient.

## Abstract (web summary)

Lung transplantation is hindered by the scarcity of organs and by mortality following primary graft dysfunction. Here, the authors show that cytokine absorption can be used in donor lungs during ex vivo lung perfusion and post-transplant, and leads to restored lung function and reduced primary graft dysfunction in animal models.

## Introduction

Organ transplantation remains a vital tool in the arsenal of treatments against end-stage disease, yet the field remains hindered by a scarcity of organs. Deemed a public health crisis, the World Health Organization remarks that just 10% of the global demand for organ transplantation is being met^1^. LTx remains limited by donor organ availability, resulting in waiting list death. In stark contrast with the 83% of potential donor kidneys which are transplanted, there are estimates that only 40% of potential donor lungs are selected for transplantation owing to donor lung injury and fear of ensuing primary graft dysfunction (PGD)^2,3^. Despite advancements in the field, PGD is still the leading cause of early mortality and contributes to the onset of chronic lung allograft dysfunction (CLAD), the foremost cause of late mortality^4–6^.

Rejected donor lungs are often deemed irreparable due to lung injury^3,7–9^. Among other causes of damage in donor lungs, such as aspiration, infection or neurogenic edema, acute lung injury (ALI), and the more severe acute respiratory distress syndrome (ARDS), stand as a frequent root of severe respiratory failure. The damage is characterized by an inflammatory injury at the alveolar capillary barrier with edema in the airspaces. ALI and ARDS involve an intense inflammatory response wherein cytokines, including interleukin-6 (IL-6), interleukin-1β (IL-1β) and tumor necrosis factor α (TNF-α), play a critical role as signaling molecules that initiate, amplify, and maintain inflammatory responses both locally and systemically^10–14^. Cytokine reduction by adsorption has been shown to have a positive impact on orthotopic heart transplantation and has reduced delayed graft function in the setting of kidney transplantation^15,16^. There is still debate on the efficacy of cytokine adsorption with reports of no provided survival benefit to patients with septic shock^17^. Cytokine adsorbers have been used to treat sepsis or ALI by reducing the levels of cytokines such as IL-6, IL-1β and TNF-α^11,18–20^. While cytokine adsorbers are being studied for their potential in ARDS patients, they have not been fully explored in the context of rescuing ARDS-damaged donor lungs for later transplantation^18,21–25^. Previous models involving transplantation have utilized healthy lungs subjected to a prolonged cold ischemic time, but there has not yet been an evaluation of cytokine adsorption in a model of ARDS-damage to the donor prior to organ retrieval^26,27^.

Ex vivo lung perfusion (EVLP) is a novel approach for assessing previously unacceptable donor lungs and has been used in the successful transplant of lungs evaluated on the EVLP system^28–31^. Cytokine adsorption has recently been evaluated in conjunction with EVLP in pre-clinical settings and has been employed as a therapy for healthy lungs subjected to extended cold ischemic storage^26,27^. Considering that the tissue was healthy at its origin, the effect of cytokine adsorption in restoring it from exposure to prolonged ischemia would allow for greater transportation times and would facilitate scheduling of operations. This would, however, differ from studying lungs that were damaged at the time of explantation with the intention of increasing the number of donor lungs viable for transplantation.

In the present study we evaluated the potential for transplanting lungs with ARDS by using cytokine adsorption in the recipient peri- and post-transplantation, with a primary endpoint of lung function as measured by the PaO_2_/FiO_2_ ratio. This treatment was administered either in two time points at both EVLP and post-transplantation via an extracorporeal cytokine hemoadsorber (entitled the two-step treatment) or solely post-transplantation (one-step treatment). Clinically relevant and molecular outcomes during EVLP and in the days following transplantation were compared to non-treated recipients. We hypothesized that a cytokine adsorber would restore lungs with ARDS and reduce the incidence of PGD.

## Results

### Overview of different treatment groups versus non-treatment group

The non-treated group refers to those lungs with lipopolysaccharide (LPS)-induced ARDS which then received EVLP and underwent transplantation without cytokine adsorption.

**Fig. 1:**
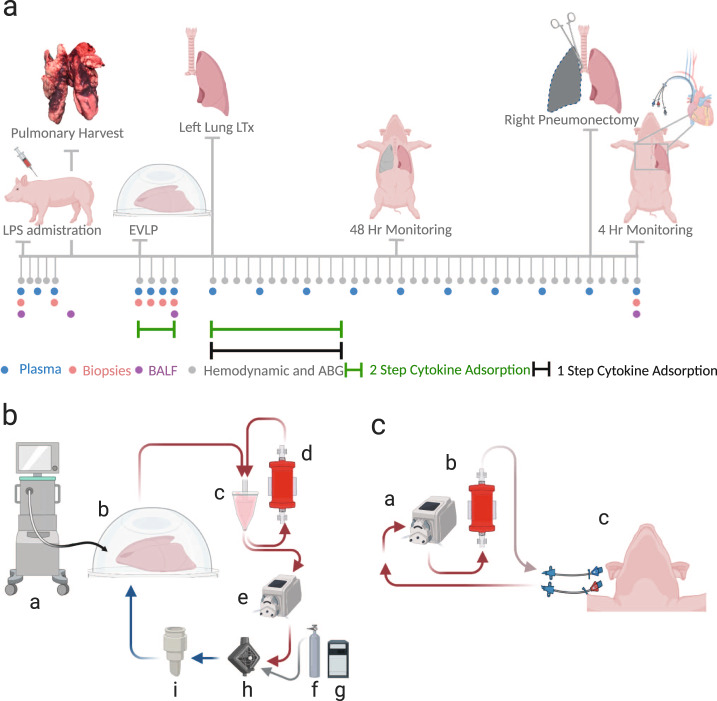
Experimental overview and technique for cytokine adsorption and lung transplantation. **a** Timeline of lipopolysaccharide (LPS)-induced acute respiratory distress syndrome (ARDS) lung injury and lung recovery by therapeutic interventions during ex vivo lung perfusion (EVLP) and transplantation (LTx) follow-up. Sample collection, including, plasma, biopsies, bronchoalveolar lavage fluid (BALF), hemodynamic measurements and arterial blood gases (AGB) and differential treatment regimens are indicated. Pulmonary harvest was conducted after confirmation of ARDS and the lungs where then placed on EVLP. The recipient was monitored for 48 hours after left lung transplantation and a mid-sternotomy followed by a right pneumonectomy in the last four hours allowed for isolated monitoring of the transplanted lung. A Swan-Ganz catheter was also placed in this monitoring period. **b** Setup of cytokine adsorption during EVLP. A mechanical ventilator (**a**) was connected to the lungs in the dome (b). Flow of perfusate continued into the reservoir (c) which fed into the cytokine adsorber (d) that then directed adsorbed perfusate back into the reservoir. Flow continued as per established methodology using a peristaltic pump (e) into a deoxygenator (h) connected to a gas supply (f) and heater (g). Following the leukocyte filter (i), the perfusate returned to the lungs. **c** Setup of cytokine adsorption post-transplantation. A veno-venous shunt using a hemodialysis catheter was inserted into the jugular vein. This facilitated flow through a pump (a) that was in line with the cytokine adsorber (b). After adsorption, flow returned to the circulation via the hemodialysis catheter in the jugular vein. Created with BioRender.com.

The treated groups were subdivided between the “one-step treated” group and the “two-step treated” group (experimental timeline demonstrated in Fig. 1a). One-step treatment refers to lungs with LPS-induced ARDS wherein EVLP was administered without cytokine adsorption and where transplantation was followed by 12 hours of extracorporeal cytokine adsorption. In the two-step treatment group, the lungs received cytokine adsorption both during EVLP and again for 12 hours of extracorporeal cytokine hemoadsorption post LTx.

### Establishment of ARDS using LPS in the Donor

Donors were treated intravenously with LPS with a dosage calculated according to weight and then monitored for development of ARDS. This follows an established model of ARDS, as previously published^32^. All LPS-treated donors developed mild to moderate ARDS within 120 ± 30 min, as defined by two separate arterial blood gases within a 15-minute interval. There was no significant difference between the severity of ARDS that ensued in donors that would go on to receive treatment (PaO_2_/FiO_2_ ratio = 208.2 ± 55.5 mmHg in the two-step treatment group; PaO_2_/FiO_2_ ratio = 204.8 ± 43.4 mmHg in the one-step treatment group) compared to those assigned to the non-treated group (ratio = 225.3 ± 33.6 mmHg, *p* = 0.733). All donors showed hemodynamic instability after LPS administration and required inotropic support. In this study, inotropic support refers to the use of agents like dobutamine, epinephrine, and norepinephrine with the clinical purpose of maintaining hemodynamic stability. Table 1 demonstrates this instability of the donors as seen in decreased oxygen saturation, decreased lung compliance, increased pulmonary vascular resistance, decreased systemic vascular resistance. Additionally, the mean arterial pressure was decreased while the cardiac output and the cardiac index increased.

**Table 1:** Clinically relevant measurements of vitals and mechanical ventilator settings during establishment of LPS-induced ARDS for all pigs.

	Baseline (*n* = 16)	Confirmed ARDS (*n* = 16)	*p* value
Sat (%)	98.9 ± 1.4	96.1 ± 3.4	>0.9999
HR (bpm)	73.8 ± 18.2	131.8 ± 18.3	0.5670
SBP (mmHg)	101.5 ± 10.2	100.6 ± 23.4	>0.9999
DBP (mmHg)	70.6 ± 10.7	62.9 ± 24.2	>0.9999
MAP (mmHg)	83.2 ± 11.0	72.6 ± 22.6	>0.9999
CVP (mmHg)	6.8 ± 2.9	6.6 ± 2.5	>0.9999
Temp (°C)	38.5 ± 1.7	39.0 ± 2.0	>0.9999
SPP (mmHg)	25.3 ± 4.8	39.6 ± 9.4	>0.9999
DPP (mmHg)	13.5 ± 4.7	26.8 ± 8.4	>0.9999
MPP (mmHg)	18.9 ± 4.1	31.6 ± 6.8	>0.9999
Wedge (mmHg)	10.6 ± 3.4	10.4 ± 5.5	>0.9999
CO (L/min)	4.0 ± 0.9	5.8 ± 2.2	>0.9999
SVR (DS/cm^5^)	1517.2 ± 312.1	1044.3 ± 405.4	**<0.0001**
PVR (DS/cm^5^)	173.2 ± 68.1	364.0 ± 183.4	**<0.0001**
CI	2.8 ± 0.5	4.3 ± 1.6	>0.9999
pH	7.4 ± 0.1	7.3 ± 0.1	>0.9999
PaCO_2_ (mmHg)	40.7 ± 6.1	54.0 ± 6.2	>0.9999
PaO_2_ (mmHg)	247.3 ± 33.6	107.8 ± 24.5	**<0.0001**
Hb (g/L)	91.2 ± 10.4	95.1 ± 13.1	>0.9999
Lactate (mmol/L)	1.6 ± 0.5	2.4 ± 1.0	>0.9999
BE (mmol/L)	4.5 ± 2.7	2.1 ± 1.5	>0.9999
MV (L/min)	7.9 ± 1.1	8.5 ± 1.6	>0.9999
Max. Pressure (cmH_2_O)	16.7 ± 2.6	20.4 ± 3.7	>0.9999
PEEP (cmH_2_O)	5.0 ± 0.0	5.0 ± 0.0	>0.9999
Vt (mL)	363.9 ± 63.0	363.4 ± 52.9	>0.9999
C_dyn_ (mL/cmH_2_O)	33.1 ± 11.6	23.8 ± 4.9	>0.9999
RR (breaths/min)	21.4 ± 3.4	23.6 ± 3.4	>0.9999
PaO_2_/FiO_2_ (mmHg)	494.2 ± 53.4	213.6 ± 43.0	**<0.0001**

*Sat* oxygen saturation, *HR* heart rate, *SBP* systolic blood pressure, *DBP* diastolic blood pressure, *MAP* mean arterial pressure, *CVP* central venous pressure, *Temp* temperature. Hemodynamic variables: *SPP* systolic pulmonary pressure, *DPP* diastolic pulmonary pressure, *MPP* mean pulmonary pressure, *Wedge* pulmonary artery wedge pressure, CO cardiac output, SVR systemic vascular resistance. Blood gas parameters: pH, *PaO*_*2*_ partial pressure of oxygen, *PaCO*_*2*_ partial pressure of carbon dioxide, *Hb* hemoglobin, lactate, *BE* base excess, *PaO*_*2*_*/FiO*_*2*_ partial pressure of oxygen divided by fraction of inspired oxygen. Mechanical ventilator settings with volume-controlled ventilation: *MV* minute volume, *PIP* peak inspiratory pressure, *PEEP* peak inspiratory pressure, positive end-expiratory pressure, *Vt* tidal volume, *Cdyn* dynamic compliance, *RR* respiratory rate, *FiO*_*2*_ fraction of inspired oxygen.

Two-sided Mann–Whitney test was used for statistical analysis. *P* values less than 0.05 are highlighted in bold text.

**Fig. 2:**
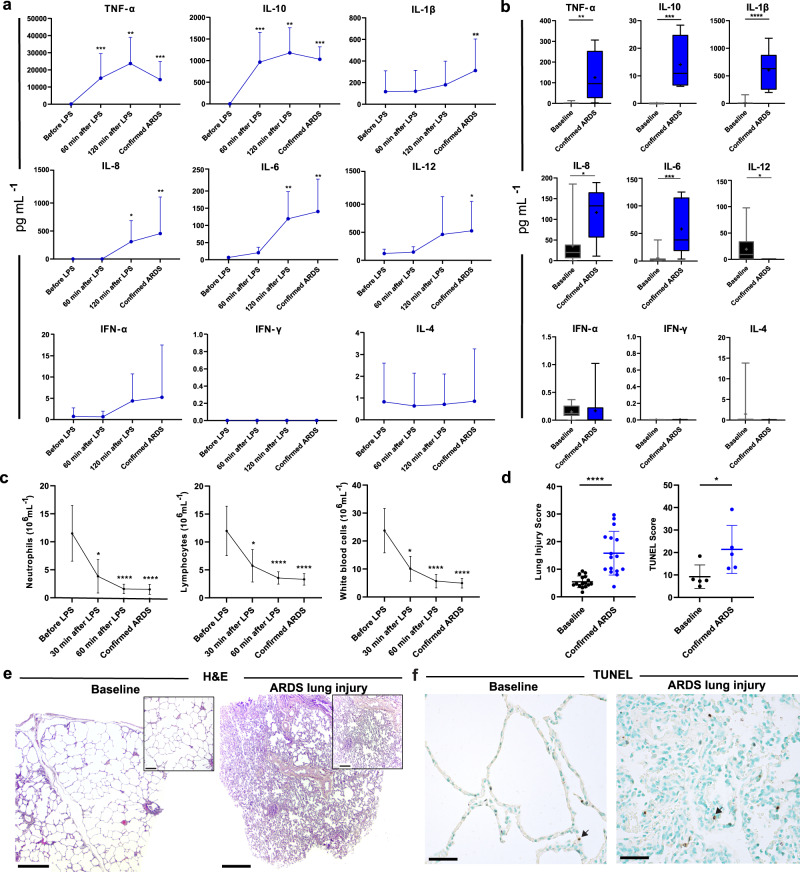
Establishment of Acute Respiratory Distress Syndrome (ARDS) lung injury in the donor. ARDS was induced via lipopolysaccharide (LPS) administration and was confirmed via two blood gases taken at a 15-minute interval with values in accordance with the Berlin definition of ARDS. **a** Cytokine measurement in plasma in the donor before LPS was administered and then 60 and 120 min after LPS was given (*n* = 12). Cytokines were also measured in the plasma at the time of confirmed ARDS. **b** Cytokine measurement in bronchoalveolar lavage fluid (BALF, *n* = 12). BALF was procured as a baseline measure before LPS administration and then again at confirmed ARDS, represented as a box-and-whiskers plot with a median line, a plus at the mean, and minimum and maximum values. **c** Neutrophils, lymphocytes, and white blood cell counts were recorded at baseline, 30 and 60 min after LPS, and at confirmed ARDS. **d** Scoring of lung injury of baseline biopsies and biopsies taken at pulmonary harvest after ARDS confirmation (left) and scoring of terminal deoxynucleotidyl transferase dUTP nick end labeling (TUNEL) positive cells/mm^2^ (right). **e** Baseline (left) and ARDS lung injury (right) hematoxylin and eosin (H&E) staining. Scale bar in the larger image represents 0.5 mm. The callout shows a magnified portion of the tissue where the scale bar represents 0.2 mm. **f** Representative images of TUNEL staining in baseline (top left) and injured lungs (bottom left) with representative black arrows indicating the type of positively stained cell counted. All graphs represent data from all donors (*n* = 16) except TUNEL (*n* = 5). Statistically significant differences between groups were tested with two-sided Student’s *T*-test and within groups with ANOVA when data were normally distributed. The two-sided Mann–Whitney test and the Kruskal–Wallis test were used when data were not normally distributed. *p < 0.05, **p < 0.01, ***p < 0.001, ****p < 0.0001. All values represent the mean ± standard deviation unless otherwise stated.

A significant increase in cytokines, including IL-1β, IL-6, IL-8, IL-10, IL-12, and TNF-α, which are known to play a critical role in ARDS, was seen during the induction of ARDS in plasma and in BALF as expected, confirming the disease model (*n* = 12, Fig. 2a, b, Supplementary Figs. 1, 4). In plasma, TNF-α values rose from a baseline value of 103.4 ± 145.3 pg/mL to 14347.0 ± 10567.0 pg/mL at the time of confirmed ARDS (*p* = 0.0001). In a similar pattern, IL-1β increased from 96.8 ± 180.7 pg/mL to 194.6 ± 273.3 pg/mL (*p* = 0.008). IL-6 increased from a baseline value of 6.7 ± 4.4 pg/mL to 140.2 ± 94.8 pg/mL at confirmed ARDS (*p* = 0.003). IL-8 rose from 0.6 ± 0.7 pg/mL to 454.2 ± 653.7 (*p* = 0.002) and IL-10 from non-detected to 1030.5 ± 288.8 pg/mL (*p* = 0.001). Lastly, an increase in IL-12 was detected from 120.7 ± 76.9 pg/mL to 525.4 ± 526.2 pg/mL (*p* = 0.007, Fig. 2a).

To investigate the relative amounts of white blood cell types, cell counts were measured every 30 minutes in the donor animals. A dramatic decrease in intravascular white blood cells following LPS administration was observed (Fig. 2c), as described previously in LPS-induced septicemia-like conditions^33^. Neutrophils decreased from 11.5 ± 5.0 × 10^6^ cells/mL at baseline to 1.5 ± 0.9 × 10^6^ cells/mL at the time of confirmed ARDS, which was a decrease also seen in lymphocytes which fell from 12.0 ± 4.4 × 10^6^ cells/mL to 3.3 ± 1.0 × 10^6 ^cells/mL.

Lung tissue taken before LPS administration for histological analysis appeared normal, with no anomalies (Fig. 2d, e). Following the administration of LPS, and as previously observed using a similar model of ARDS-induction^32^, there was a significant infiltration of the alveolar spaces by immune cells and notable atelectasis which affected the majority of alveolar spaces. Erythrocytes were also found in abundance within the alveolar space with the occasional appearance of early hyaline membrane formation. Furthermore, vasodilation was visible within the capillaries where there was also hemorrhage and aggregation of neutrophils. In addition to subjective analysis of lung histology, blinded scoring was performed on all pigs at baseline, post-ARDS, post-EVLP and post-transplantation by three independent observers. Significant increases in a cumulative lung injury score from baseline were observed, accounting for multiple signs of lung injury following ARDS onset via LPS administration (baseline mean score = 5.4 ± 2.1, confirmed ARDS mean score  = 15.8 ± 7.9, *p* < 0.0001, Fig. 2d). No significant differences were seen between the treated (two-step and one-step treatment) and non-treated groups at baseline (two-step treated mean score of 4.4 ± 1.8, one-step treatment 5.5 ± 2.3 non-treated mean score of 6.1 ± 2.3, *p* = 0.41). A greater detailed figure of histology can be found in Supplementary Fig. 5. TUNEL scoring of positive cells in baseline biopsies and biopsies taken at pulmonary harvest after ARDS confirmation showed significant differences, in line with late apoptosis (baseline mean score = 9.2 ± 5.3 cells/mm^2^, confirmed ARDS mean score = 21.4 ± 10.7 cells/mm^2^, *p* = 0.03, Fig. 2d, f, greater detail in Supplementary Fig. 11). Following confirmation of ARDS, lungs were harvested en bloc approximately 1 hour after confirmed ARDS.

### Recovery of pulmonary function and inflammation following cytokine adsorption in EVLP

**Fig. 3:**
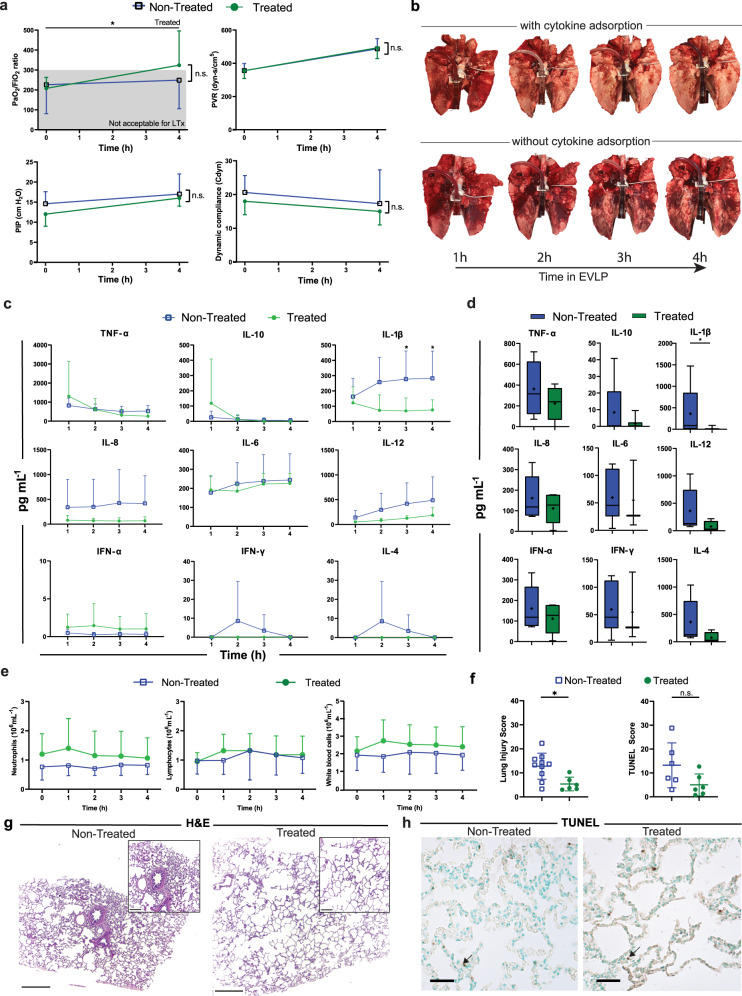
Improvement of pulmonary function and inflammation following cytokine adsorption during ex vivo lung perfusion (EVLP) treatment. Donor lungs were connected to an EVLP circuit and assigned to either the non-treated group or treated group which consisted of continuous cytokine adsorption during the four hours of EVLP. **a** Measures of pulmonary gas exchange including the PaO_2_/FiO_2_ ratio, the pulmonary vascular resistance (PVR), peak inspiratory pressure (PIP), and dynamic compliance were recorded throughout EVLP. **b** Gross morphology of the treated lungs (top) and the non-treated lungs (bottom) throughout the 4-hour period. **c** Cytokines in plasma with samples taken every hour of EVLP, with 1 hour marking the time elapsed since the start of treatment (*n* = 6 per group). **d** The bronchoalveolar lavage fluid (BALF) was tested at the end of EVLP for cytokine levels (*n* = 6 per group). **e** Cell counts of neutrophils, lymphocytes, and white blood cells were measured every hour. Data is represented as a box-and-whiskers plot with a median line, a plus at the mean, and minimum and maximum values. **f** The scores of the histologycompare cytokine adsorption groups (left) and the cell counts per mm^2^ after terminal deoxynucleotidyl transferase dUTP nick end labeling (TUNEL) staining (right). **g** Images representative of *n* = 16 samples of hematoxylin and eosin (H&E) histology of non-treated (left) lungs and treated (right) lungs. Scale bar in the larger image represents 0.5 mm. The callout shows a magnified portion of the tissue where the scale bar represents 0.2 mm. **h** Representative images of *n* = 5 lungs of TUNEL staining in non-treated (left) and treated lungs (right) with representative black arrows indicating the type of positively stained cell used in the TUNEL score. Scale bars represent 50 µm. All graphs represent data from either the treated donor lungs (*n* = 6) or non-treated lungs (*n* = 10 as the one-step group did not receive cytokine adsorption during EVLP), except for the multiplex cytokine and chemokine and the TUNEL analysis (treated donor lungs (*n* = 6) or non-treated lungs (*n* = 6)). Statistically significant differences between non-treated and treated groups were tested with two-sided Student’s *T*-test and within groups with ANOVA when data were normally distributed. The two-sided Mann–Whitney test and the Kruskal–Wallis test were used when data were not normally distributed. *p < 0.05, **p < 0.01, ***p < 0.001, n.s. non significant. All values represent the mean ± standard deviation unless otherwise stated.

To mimic clinical transplantation, the lungs were put in cold storage in Perfadex® PLUS solution (XVIVO perfusion, Gothenburg, Sweden) for 2 hours. After, they were connected to the EVLP for 4 hours. All pulmonary grafts had ARDS according to the Berlin ARDS definition using the PaO_2_/FiO_2_ ratio at the initiation of EVLP^34^. In this definition, the distinguishing criteria of ARDS severity is the PaO_2_/FiO_2_ ratio with a PEEP of at least 5 cmH_2_O such that mild ARDS falls in a PaO_2_/FiO_2_ ratio between 201–300 mmHg, moderate between 101–200 mmHg, and severe as ≤100 mmHg. Over the course of EVLP, the cytokine adsorber-treated lungs had an increase in gas exchange capacity and reached a PaO_2_/FiO_2_ ratio of 324 ± 70. This represented a significant change over time (*p* = 0.03) and met the threshold of clinical acceptance for transplantation. The non-treated lungs did not pass clinical acceptance with a PaO_2_/FiO_2_ ratio of 249 ± 143 in the 10 pigs assigned to the non-treated group and a ratio of 228 ± 120 in the one-step treatment group (Fig. 3a, Supplementary Fig. 14a). No significant differences could be seen between either of the groups. All lungs increased in PVR during the EVLP, however this increase was not significant within each group or between the groups (Fig. 3a, Supplementary Fig. 14a). There were no significant differences between the groups in airway pressure or the pulmonary compliance (Fig. 3a, Supplementary Fig. 14a). This change in pulmonary function is reflected on a macroscopic level, as the gross morphology of the treated lungs also showed decreased hemorrhage compared to non-treated donor lungs (Fig. 3b).

All groups required additional Steen solution in the EVLP circuit over the 4 hours. Two lungs in the non-treated group, two in the two-step treated group, and one in the one-step treated group had acute pulmonary edema after 2 hours in EVLP with fluid withdrawal in the main bronchus. The addition of supplementary Steen solution, a procedural measure taken when perfusate levels dropped below 300 mL in the EVLP reservoir, is a sign of edema in the lungs.

To monitor the inflammatory response during EVLP and see if changes in perfusate markers coincided with signs of restored pulmonary function, cytokine concentrations were measured in the perfusate each hour (Fig. 3c, Supplementary Fig. 2). A significant decrease in the proinflammatory cytokine IL-1β was seen in the cytokine adsorbed lungs relative to the non-treated lungs (*n* = 6) in the perfusate and BALF. This is exemplified by the 4th hour of EVLP which showed a decrease from 282.9 ± 177.4 pg/mL in the non-treated perfusate to 75.6 ± 66.4 pg/mL in the treated perfusate (Fig. 3c) and from 362.4 ± 626.7 pg/mL in non-treated BALF to 15.3 ± 37.4 pg/mL in two-step treated BALF (*p* = 0.0216, Fig. 3d). A generally lower level of cytokines could be detected in the treated lungs, however none reached significance (Fig. 3c, d). During EVLP, the cell counts in the perfusate were measured every hour and there was no significant change in the number of neutrophils, leukocyte, and total white blood count within the 4-hour period in all donor groups (Fig. 3e, Supplementary Fig. 14b).

Following EVLP, the histology of the tissue showed a significant difference between the cytokine adsorption-treated lungs and those without adsorption with regard to morphology and lung injury (Fig. 3f, g). A greater detailed figure of histology can be found in Supplementary Fig. 6. Treated lungs had fewer immune cells, erythrocytes, and atelectasis compared to lungs without adsorption. Histological scoring revealed a significant improvement towards healthy lung tissue between the treated (average score of 5.4 ± 2.8) and the non-treated donors (score of 12.8 ± 5.4, *p* = 0.016) following EVLP (Fig. 3f). According to the wet/dry ratio, an index of accumulated fluid in the lungs, there was no significant difference between the groups at the end of EVLP (Supplementary Fig. 15, *p* = 0.07).

To evaluate the incidence of late apoptosis within each group, TUNEL staining was performed and showed no difference between the treated and non-treated groups. Those with cytokine adsorption in EVLP had a mean value of 5.0 ± 4.6 cells/mm^2^ while those who were non-treated measured 13.2 ± 9.4 cells/mm^2^ (*p* = 0.13, Fig. 3f, h, enlarged images in Supplementary Fig. 12).

### Restoration of pulmonary function and reduced inflammation after 48 hours of transplantation in treated recipients

After 4 hours of EVLP, the lungs were subsequently transplanted. A left thoracotomy was followed by a left pneumonectomy so that a left LTx could occur. Once the lung was transplanted, extracorporeal hemoperfusion with a cytokine adsorber in the circuit was placed in the treated recipients (one-step treatment and two-step treatment) for the first 12 postoperative hours (Fig. 1c, Supplementary Video 1). Both treated groups required significantly less inotropic support post-transplantation, showing improved hemodynamic stability compared to the non-treated group. Hemodynamic parameters, blood gas values, and mechanical ventilator settings are shown in Table 2.

**Table 2:** Clinically relevant measurements of vitals and mechanical ventilator settings post transplantation for all recipients.

Time	Sat (%)	HR (bpm)	SBP (mmHg)	DBP (mmHg)	MAP (mmHg)	CVP (mmHg)	Temp (°C)	pH	BE (mmol/L)	MV (L/min)	Max. Pressure (cmH_2_O)	PEEP (cmH_2_O)	Vt (mL)	Cdyn (mL/ cmH_2_O)	RR (breaths/min)
**Baseline**	98.2 ± 1.4	67.3 ± 14.8	120.3 ± 17.8	87 ± 14.3	100 ± 14.7	8.3 ± 4.4	37.1 ± 1	7.5 ± 0.08	6.2 ± 3.2	8.35 ± 1.5	20.8 ± 3.3	5.8 ± 2.0	396.2 ± 33.5	26.4 ± 7.7	21.5 ± 3.8
	**100** **±** **0**	**93.7** **±** **15**	**94.5** **±** **9.3**	**58.2** **±** **9.5**	**72** **±** **10.8**	**8** **±** **3.2**	**39.7** **±** **1.3**	**7.4** **±** **0.04**	**4.2** **±** **3.8**	**7.3** **±** **0.2**	**19.7** **±** **2.8**	**5.5** **±** **0.5**	**361.9** **±** **51.2**	**30.1** **±** **8.2**	**25.2** **±** **0.5**
	98.3 ± 2.7	74.0 ± 16	105.3 ± 10.4	69.5 ± 7.8	83.5 ± 8.6	5.3 ± 3.5	36.8 ± 1.5	7.5 ± 0.2	5.6 ± 2.1	8.5 ± 2.0	17.7 ± 1.5	5.0 ± 0.0	388.3 ± 44.3	31.8 ± 5.4	20.7 ± 3.3
**1** **h**	97.2 ± 2.4	88.7 ± 3.8	101.2 ± 12.4	62 ± 13	76.7 ± 14.5	5.8 ± 2.4	36.6 ± 1.9	7.3 ± 0.06	4.3 ± 3.5	10 ± 1.3	23.6 ± 2.6	6 ± 2.0	396.7 ± 53.9	24.3 ± 1.4	25.8 ± 3.9
	**96.5** **±** **3.8**	**90.8** **±** **6.8**	**102** **±** **8.5**	**62.7** **±** **7.5**	**79.25** **±** **6.3**	**6.2** **±** **3.5**	**40.7** **±** **0.8**	**7.3** **±** **0.05**	**3.3** **±** **3**	**7.7** **±** **0.6**	**27.2** **±** **4**	**7.7** **±** **1.5**	**413.0** **±** **59**	**24.2** **±** **2.7**	**28** **±** **2.8**
	97.7 ± 1.7	90.8 ± 6.2	102.8 ± 4.1	67.5 ± 5	87.3 ± 5.5	4 ± 3.2	36.4 ± 2.4	7.3 ± 0.04	2.4 ± 1.3	9.3 ± 1.7	22.5 ± 2	5.8 ± 1.1	387.8 ± 38	24.3 ± 4.0	22.5 ± 2.5
**12** **h**	97.7 ± 1.9	77.3 ± 15.3	106.3 ± 8.6	70 ± 8.7	84.5 ± 9.2	8 ± 3.9	38.5 ± 1.2	7.4 ± 0.04	5.5 ± 3.2	9.8 ± 1.5	22.5 ± 2	6.5 ± 2	404.8 ± 56.3	30.2 ± 3.4	27 ± 3
	**100** **±** **0**	**95.5** **±** **11.6**	**109** **±** **12.1**	**66** **±** **6.2**	**84.2** **±** **8.7**	**5.7** **±** **2.6**	**38.5** **±** **0.9**	**7.4** **±** **0.04**	**5.6** **±** **1**	**8.05** **±** **0.5**	**25.5** **±** **2.6**	**7.2** **±** **0.5**	**443.2** **±** **40.3**	**20.9** **±** **4.2**	**26.7** **±** **1.5**
	97.8 ± 3.3	76.4 ± 17.3	103.2 ± 4.9	75 ± 19.1	82.4 ± 7.9	5 ± 2.8	38.7 ± 1	7.4 ± 0.05	3.7 ± 2.2	9.7 ± 1.7	20.2 ± 1.7	6.4 ± 1.5	408.6 ± 31.4	30.1 ± 2.8	23.23.6
**24** **h**	98.2 ± 1	80.4 ± 13.2	107.8 ± 5.2	63.8 ± 5	79.6 ± 5.9	7.4 ± 2	38.5 ± 1.1	7.5 ± 0.1	6.6 ± 2.6	11 ± 0.8	22 ± 1.4	6.2 ± 2.1	418.3 ± 58	30.7 ± 12.9	24.5 ± 2.3
	**99.7** **±** **0.5**	**108.7** **±** **5.1**	**113.5** **±** **16**	**70.5** **±** **20**	**88.7** **±** **18.3**	**8.7** **±** **4.6**	**40.4** **±** **0.4**	**7.4** **±** **0.05**	**5.1** **±** **1.6**	**8.05** **±** **0.4**	**25** **±** **2.8**	**7.2** **±** **0.5**	**427.0** **±** **46.3**	**27.5** **±** **2.8**	**26.7** **±** **1.5**
	97.2 ± 2	73.6 ± 15	100.8 ± 7.8	72.4 ± 16.8	79.2 ± 9.8	5.4 ± 2.9	39.3 ± 0.5	7.4 ± 0.08	7.5 ± 1.9	9.3 ± 0.8	20 ± 1.8	6.4 ± 1.5	413.6 ± 52.6	30.6 ± 3.5	22.6 ± 3.1
**36** **h**	97.8 ± 2	74.2 ± 14.3	108 ± 6.2	67 ± 4.9	84 ± 5.5	7 ± 2.3	38.7 ± 1.1	7.4 ± 0.04	6 ± 2.4	10 ± 1	22 ± 1.7	6 ± 3.5	417 ± 48.4	22.8 ± 3.3	26.3 ± 2.8
	**99.6** **±** **0.5**	**112.3** **±** **2.8**	**114.3** **±** **12.8**	**74.6** **±** **20.1**	**91.6** **±** **17.7**	**9.3** **±** **4**	**40.7** **±** **0.3**	**7.4** **±** **0.07**	**6.5** **±** **0.9**	**7.7** **±** **0.8**	**26.3** **±** **3.7**	**7.6** **±** **0.5**	**432.9** **±** **38.9**	**27.2** **±** **4.2**	**26.7** **±** **1.5**
	97.6 ± 3.6	75.8 ± 14.7	108.6 ± 9.4	75 ± 18.6	85 ± 11	6 ± 3.5	39.4 ± 0.4	7.4 ± 0.16	8.8 ± 1.2	10 ± 1.6	20 ± 2.0	6.4 ± 1.9	417 ± 48.1	30.2 ± 3.1	22.6 ± 2.6
**48** **h**	97.6 ± 1	74.2 ± 13.2	102.4 ± 12.7	66 ± 12.2	82.8 ± 7.9	6.4 ± 2.7	38.8 ± 1.1	7.5 ± 0.2	6.0 ± 0.4	10 ± 1	23 ± 1.7	6.5 ± 3.5	426.7 ± 10.6	22.7 ± 1.2	25.8 ± 2.8
	**99** **±** **1.7**	**110** **±** **9.6**	**104** **±** **6**	**63.3** **±** **11.2**	**78.6** **±** **16.4**	**8.6** **±** **3.5**	**40.5** **±** **0.4**	**7.4** **±** **0.07**	**6.5** **±** **0.9**	**7.5** **±** **1.2**	**27.5** **±** **4.4**	**8** **±** **0.8**	**427.0** **±** **46.3**	**25.9** **±** **6.9**	**26.7** **±** **1.5**
	97.2 ± 3.6	75.8 ± 14.7	103.2 ± 6.7	71 ± 19.7	80.8 ± 15.6	5.8 ± 2	39.3 ± 0.5	7.4 ± 0.14	6.5 ± 2.8	9.5 ± 1	21 ± 2.2	6.4 ± 1.5	412 ± 51	30 ± 3.5	22.6 ± 2.6

The mean ± SD values for two-step treated animals (*n* = 6) are shown in the first row and one-step treated animals (*n* = 4) in the second row with bold text while the non-treated animals (*n* = 6 until 12 h, *n* = 5 after 12 h) are shown in the third row for each respective timepoint.

Two-step treated: First rows; One-step treated: Second rows, bold text; Non-treated: Third rows

*Sat* Measurements for oxygen saturation, *HR* heart rate, *SBP* systolic blood pressure, *DBP* diastolic blood pressure, *MAP* mean arterial pressure, *CVP* central venous pressure, *Temp* temperature, pH, *BE* base excess, Mechanical ventilator settings with volume-controlled ventilation: *MV* minute volume, *PIP* peak inspiratory pressure, *PEEP* peak inspiratory pressure, positive end-expiratory pressure, *Vt* tidal volume, *RR* respiratory rate.

**Fig. 4:**
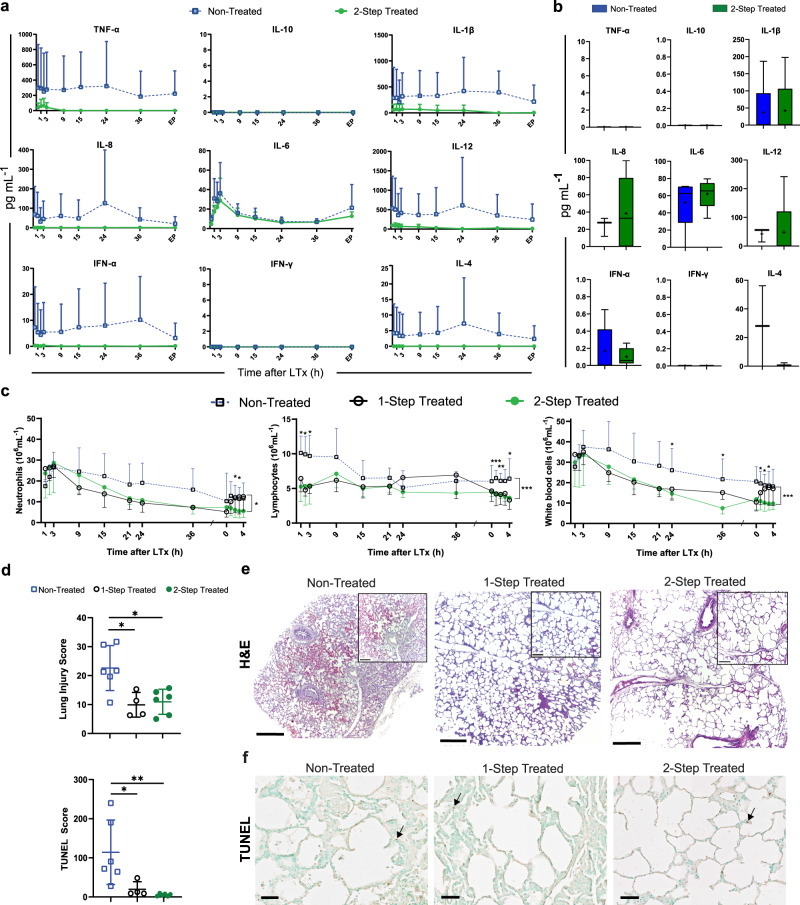
Reduced inflammatory state during lung transplantation (LTx) and follow-up. **a** Plasma cytokine levels were monitored throughout the 48-hour period following transplantation, with 1 h marking the time elapsed since the start of treatment. EP stands for endpoint of the experimental timeline. **b** Bronchoalveolar lavage fluid (BALF) was tested for cytokine concentrations at the termination of the experiment represented as a box-and-whiskers plot with a median line, a plus at the mean, and minimum and maximum values. **c** Cell counts including neutrophils, lymphocytes, and white blood cells were analyzed. Statistical significance applies to direct comparison of two-step treatment to the non-treated group. **d** Scoring of the lung injury across groups (top) and scoring of the terminal deoxynucleotidyl transferase dUTP nick end labeling (TUNEL) cell counts per mm^2^ (bottom). **e** Hematoxylin and eosin (H&E) staining representative of *n* = 16 of non-treated (left), one-step treated (middle) and two-step treated (right) biopsies taken at the end, following 4 h of isolated transplanted lung function. **f** Representative images of *n* = 5 lungs of TUNEL staining in non-treated (left), one-step treated (middle) and two-step treated lungs (right) with representative black arrows indicating positively stained cells used in the TUNEL score. Scale bars represent 50 µm, respectively. All graphs represent data from either the two-step treated recipient lungs (*n* = 6), the one-step treated recipients (*n* = 4) or non-treated lungs (*n* = 6). Statistically significant differences between groups were tested with two-sided Student’s *T*-test and within groups with ANOVA when data were normally distributed. The two-sided Mann–Whitney test and the Kruskal–Wallis test were used when data were not normally distributed. *p < 0.05, **p < 0.01, ***p < 0.001. All values represent the mean ± standard deviation unless otherwise stated.

To track the inflammatory response post-transplantation, cytokine concentrations were measured in plasma and collected at multiple time points post-transplantation. All measured cytokines were generally decreased post-transplantation in the cytokine adsorber-treated group, however none of these reached statistical significance (Fig. 4a, b, Supplementary Fig. 3). When looking at the count of blood cells post-transplantation, significant decreases in both neutrophil counts and total white blood cell counts were observed in the two-step treated group in the latter hours of transplantation follow-up, especially after the right pneumonectomy (Fig. 4c). At the end of observation, neutrophils were reduced from 11.6 ± 3.0 × 10^6^ cells/mL (non-treated) to 5.6 ± 1.3 × 10^6^ cells/mL (two-step treated) but were unchanged in the one-step treated (12.5 ± 4.0 × 10^6^ cells/mL). Total white cell counts dropped from 18.1 ± 3.4 × 10^6^  cells/mL (non-treated) to 9.8 ± 1.2 × 10^6^  cells/mL (two-step) and were unchanged in the one-step treated (17.0 ± 4.7 × 10^6^ cells/mL). Furthermore, lymphocyte counts were significantly lower in the treated groups at the majority of time points studied, showing a decrease from 6.4 ± 1.2 (non-treated) to 3.7 ± 0.2 (two-step treated), and 3.3 ± 0.78 × 10^6^ cells/mL (one-step treated) after 4 hours following right pneumonectomy (Fig. 4c).

The lung tissue wet/dry weight ratios were measured after 4 hours of EVLP, and 48 hours post-transplantation in the two-step treatment and non-treatment group. While the ratios between the treated and non-treated group were not statistically significant, there was a significant decrease in the cytokine adsorption treated lungs’ average wet/dry ratio from the time of EVLP to the end of LTx (Supplementary Fig. 15). The average wet/dry ratio in the left lower lobe after 4 hours of EVLP was 8.55 ± 1.8 while it was 6.1 ± 0.8 48 hours following transplantation in the two-step treated group (*p* = 0.0196).

With regard to histology, in the non-treated group, there were significant morphological changes characteristic of ARDS which remained, including the accumulation of immune cells, intra-alveolar hemorrhage, and the collapse of most alveolar spaces after transplantation, which was reflected in the scoring (Fig. 4d, e). In the one-step and two-step treated recipients, an immune response was still seen, exemplified by the infiltration of immune cells, as expected in transplanted lungs, but the alveolar spaces were mostly open, and respiratory bronchioles and blood vessels appeared without major visible damage. There was a decreased score in both treated lungs relative to non-treated ones 48 hours post-transplantation (score of 9.92 ± 4.27 in the one-step treatment; score of 10.94 ± 4.33 in the two-step treatment; score of 22.61 ± 7.76 in the non-treated group). The two-step treated group and one-step treated group had significantly lower scores compared to the non-treated group (*p* = 0.026 and *p* = 0.0381 respectively, Fig. 4d). Greater detailed figures of histology can be found in Supplementary Fig. 7. TUNEL staining showed greater numbers of apoptotic cells normalized to tissue area in the histological section from non-treated recipients (114.2 ± 82.3 cells/mm^2^) compared to the one-step treated group (19.7 ± 19.2 cells/mm^2^) and the two-step treated group (5.5 ± 3.0 cells/mm^2^). A significant difference was seen between the non-treated and the one-step treated group (*p* = 0.027), and also between the non-treated and the two-step treated group (*p* = 0.006), Fig. 4d, f, Supplementary Fig. 13).

### Reduced Primary Graft Dysfunction (PGD) in Treated Recipients

**Fig. 5:**
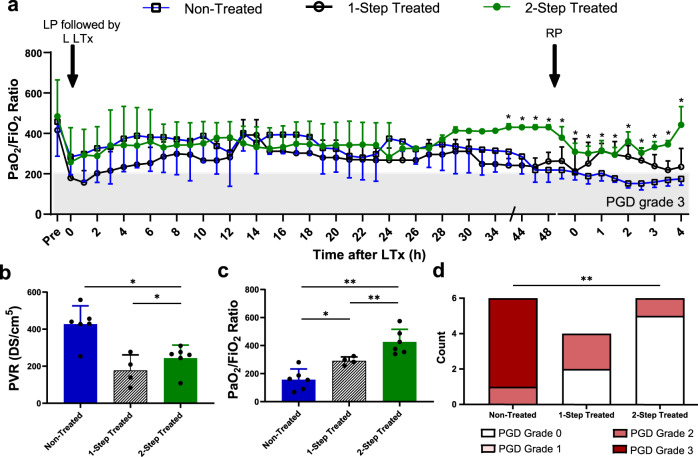
Reduced primary graft dysfunction (PGD) in treated recipients. **a** PaO_2_/FiO_2_ ratios for all groups were followed from before transplantation in the recipient to 48 h of follow-up. The first arrow indicates a left pneumonectomy followed by left lung transplantation (LP followed by L LTx) and the second arrow depicts the time of right pneumonectomy (RP). Statistical significance applies to direct comparison of two-step treatment to the non-treated group **b** Pulmonary vascular resistance (PVR) data (left) and **c** PaO_2_/FiO_2_ ratios (right) for all groups at the end of the experiment including all recipients. **d** Comparison of PGD grades following transplantation. All graphs represent data from either the two-step treated recipient lungs (*n* = 6), the one-step treated recipient (*n* = 4) or non-treated lungs (*n* = 6, *n* = 5 following 9 h post transplantation). Statistically significant differences between groups were tested with two-sided Student’s *T*-test and within groups with ANOVA when data were normally distributed. The two-sided Mann–Whitney test and the Kruskal–Wallis test were used when data were not normally distributed. Chi-squared analysis was performed to analyze observed frequencies of categorical PGD grades. *p < 0.05, **p < 0.01, ***p < 0.001. All values represent the mean ± standard deviation.

All treated and non-treated recipients were monitored for 48 hours post-transplantation, with the last 4 hours encompassing isolated transplanted left lung function following a right pneumonectomy. The overview of clinically relevant vital measurements during these 4 hours are shown in Table 3 and Fig. 5, which demonstrates improved oxygenation capacity of the lung and improved pulmonary vascular resistance upon reliance on the transplanted lung alone. The pulmonary compliance was generally improved in the two-step treated group compared to the non-treated group (*p* = 0.001, Tables 2 and 3). The lactate was lower in the two-step treated group (*p* = 0.001, Table 3). During the first day post-transplantation, no significant difference could be seen in the gas exchange between the groups (Fig. 5a, *p* = 0.122). During the second day, however, and especially after the right pneumonectomy, a significant increase in gas exchange could be seen in the two-step treated compared to the non-treated group (Fig. 5a, *p* < 0.0001). At the end of the experiment (including all recipients), PVR was found to be significantly lower in both the one-step and two-step treated groups (Fig. 5b, *p* = 0.0190 and *p* = 0.0260, respectively). The PaO_2_/FiO_2_ ratios also demonstrated better outcomes with higher ratios found in both the one-step and two-step groups at the end of the experiment (Fig. 5c, *p* = 0.0190 and *p* = 0.0022, respectively). In the non-treated group, five recipients developed PGD grade 3, while one developed PGD grade 2. In the two-step treated group, only one recipient developed PGD grade 2 while the rest of the treated recipients had PGD grade 0. In the one-step treated group, two had PGD grade 0 and 2 had PGD grade 2. This represented a significant difference in the number of recipients developing PGD in either treatment group (*p* = 0.006, Fig. 5d).

**Table 3:** Overview of clinically relevant measurements of vitals and mechanical ventilator settings during the last phase of the experiment.

	Before Pneumonectomy	4 h Post Pneumonectomy	Non- vs 1-Step Treated	Non- vs 2-Step Treated	1-Step vs 2-Step Treated
Sat (%)	96.1 ± 3.3	96 ± 2.5	0.9936	0.9998	0.9952
	**100** **±** **0**	**100** **±** **0**			
	96.8 ± 1.5	95.2 ± 4.3			
HR (bpm)	91 ± 17.2	83.3 ± 19.0	0.5930	0.9857	0.4750
	**107** **±** **10.5**	**133.3** **±** **12.5**			
	79 ± 17.0	89.8 ± 34.2			
SBP (mmHg)	106.6 ± 13.1	101.6 ± 7.5	0.9931	0.9961	0.9992
	**102** **±** **8.2**	**100** **±** **9.8**			
	109.4± 8.5	105 ± 12.5			
DBP (mmHg)	63 ± 19.0	47.4 ± 17.5	0.9705	0.8935	0.9819
	**61.2** **±** **9.5**	**55.2** **±** **8.4**			
	72 ± 8.6	65.6 ± 3.1			
MAP (mmHg)	80.2 ± 20.1	64.8 ± 21.1	0.9783	0.9206	0.9866
	**73.7** **±** **12.6**	**71.5** **±** **11.6**			
	87.8 ± 11.4	80.4 ± 6.1			
CVP (mmHg)	6.8 ± 2.3	7 ± 2.5	0.9951	0.9997	0.9969
	**8.7** **±** **2.8**	**10.2** **±** **2.8**			
	7.6 ± 3.4	6 ± 3.1			
Temp (°C)	38.6 ± 0.6	38 ± 0.8	>0.9999	>0.9999	>0.9999
	**39.4** **±** **0.4**	**38.2** **±** **0.6**			
	39.5 ± 0.4	38.4 ± 1.2			
CO (L/min)	4.3 ± 0.9	3.7 ± 0.6	0.9997	0.9991	>0.9999
	**4.75** **±** **0.8**	**4.2** **±** **0.8**			
	4.5 ± 0.1	5.3 ± 0.9			
SVR (DS/cm^5^)	1327 ± 356	1415 ± 413	**0.0009**	**<0.0001**	**<0.0001**
	**1202.5** **±** **452.2**	**1192.5** **±** **240.6**			
	1180 ± 200	1030 ± 139			
pH	7.4 ± 0.1	7.3 ± 0.1	>0.9999	>0.9999	>0.9999
	**7.4** **±** **0.05**	**7.4** **±** **1.0**			
	7.4 ± 0.1	7.3 ± 0.2			
Hb (g/L)	72 ± 10.2	69.4 ± 10.5	0.7691	0.9425	0.9115
	**51.5** **±** **4.2**	**51.8** **±** **2**			
	83 ± 3.4	82.6 ± 10.1			
BE (mmol/L)	6.4 ± 3.8	8.7 ± 2.7	0.9956	>0.9999	0.9945
	**6.6** **±** **2.8**	**4.4** **±** **1.2**			
	6.9 ± 4.1	8.4 ± 1.8			
MV (L/min)	9.7 ± 1.8	11.8 ± 3.0	0.9958	>0.9999	0.9952
	**7.3** **±** **1.2**	**7.8** **±** **0.8**			
	10.1 ± 1.5	11.7 ± 1.6			
Max. Pressure (cmH_2_O)	22 ± 2.5	24 ± 3.0	0.9991	0.9993	0.9969
	**26.7** **±** **2.2**	**27.2** **±** **1.8**			
	22.4 ± 3.4	25.4 ± 2.9			
PEEP (cmH_2_O)	6.4 ± 2.2	6.0 ± 2.2	0.9975	>0.9999	0.9980
	**8** **±** **0.8**	**8.6** **±** **1**			
	6.2 ± 1.6	5.6 ± 1.3			
Vt (mL)	407.0 ± 58.5	396.5 ± 35.4	0.7573	0.9575	0.8829
	**416.5** **±** **36.8**	**416.9** **±** **55.8**			
	429.0 ± 52.5	385.2 ± 22.5			
Cdyn (mL/cmH_2_O)	26.4 ± 1.8	22.7 ± 2.4	0.9956	0.9982	0.9991
	**26.0** **±** **5.8**	**24.4** **±** **4.8**			
	27.1 ± 5.0	20.4 ± 3.4			
RR (breaths/min)	26.4 ± 4.1	22.7 ± 3.3	>0.9999	0.9830	0.9863
	**26** **±** **1.4**	**29.5** **±** **4**			
	23.8 ± 4.0	29.8 ± 4.9			
SPP (mmHg)	27 ± 6	38 ± 3	0.9989	>0.9999	0.9988
	**30** **±** **6**	**36** **±** **6.7**			
	26 ± 4	38 ± 8			
DPP (mmHg)	18 ± 11	23.5 ± 3	0.9705	0.9948	0.9874
	**16** **±** **2.9**	**17** **±** **4.8**			
	15 ± 6.0	27.4 ± 12.0			
MPP (mmHg)	24 ± 2.0	31 ± 2	0.9849	0.9997	0.9791
	**20.7** **±** **4.3**	**22.6** **±** **5.9**			
	24 ± 2.9	30 ± 8.2			
Lactate (mmol/L)	1.2 ± 0.3	1.3 ± 0.2	>0.9999	>0.9999	>0.9999
	**0.9** **±** **0.3**	**1.5** **±** **0.2**			
	1.3 ± 0.4	1.2 ± 0.4			

The values for the two-step treated recipients (*n* = 6) are shown in the first row, one-step treated recipients (*n* = 4) in the second row with bold text, and the non-treated recipients (*n* = 5) are in the third row for each respective parameter. Mann–Whitney and Kruskal–Wallis tests were used for statistical analysis. *P* values less than 0.05 are highlighted in bold text.

Two-step treated: First rows (n = 6); One-step treated: Second rows, bold text (*n* = 4); Non-treated: Third rows (*n* = 5).

*Sat* oxygen saturation, *HR* heart rate, *SBP* systolic blood pressure, *DBP* diastolic blood pressure, *MAP* mean arterial pressure, *CVP* central venous pressure, *Temp* temperature, Hemodynamic variables: *SPP* systolic pulmonary pressure, *DPP* diastolic pulmonary pressure, *MPP* mean pulmonary pressure, *CO* cardiac output, *SVR* systemic vascular resistance, *PVR* pulmonary vascular resistance, Blood gas parameters: *Hb* hemoglobin, lactate, *BE* base excess, Mechanical ventilator settings with volume-controlled ventilation: *MV* minute volume, *PIP* peak inspiratory pressure, *PEEP* peak inspiratory pressure, positive end-expiratory pressure, *Vt* tidal volume, *RR* respiratory rate.

## Discussion

This current study explores the implementation of a cytokine adsorber in the treatment of ARDS-damaged lungs, rendering the organs suitable for transplantation. The results suggest that the use of a cytokine adsorber (i) recovers pulmonary function and inflammation during EVLP, (ii) restores pulmonary function and reduces inflammation in the 48-hour follow-up post-transplantation, and (iii) was correlated with a decreased incidence of PGD in recipients. The value of this technique lies in the interest in the field in restoring injured lungs. Previous results by Hozain et al. have shown that cross-circulation may treat injured human lungs^35^, however, the implementation of xenogeneic or allogenic cross-circulation may prove challenging in practical application. In contrast, the use of EVLP is an already established method and alone can reduce acute injury in the lungs. In conjunction with a cytokine adsorber, EVLP is capable of treating healthy lungs subjected to extended cold ischemic storage^26,36^. This method has not, however, examined the results of using cytokine adsorption on lungs damaged by ARDS which are then transplanted and examined for primary graft dysfunction (PGD), the gold standard for evaluating the efficacy of potential methods in the clinic.

To address this, donor lungs with an LPS-induced ARDS injury were transplanted and treated with cytokine adsorption. LPS is derived from the outer membrane of gram-negative bacteria and upon intravenous administration, results in damage to the endothelial cells in the lung. The destruction is further driven by a systemic inflammatory response^37^. The bacterial toxin is known to interact with the endothelial lining of the vessel walls of the lung to induce programmed cell death which is thought to be central to sepsis pathogenesis^37^. This form of inducing ARDS has been studied in large animal models given the clinical translational potential of the disease. Other forms of provoking ARDS, including repeated lavage and oleic acid, result in lung pathology but suffer from the use of mechanisms that are different from those found in human patients with ARDS^38^. Advantages of the use of endotoxin include a pathophysiology similar to clinical ARDS along with technical reproducibility. Using an LPS-induced ARDS model represents an opportunity to explore the expansion of the donor pool considering the large number of organs that are rejected due to acute lung injury. ARDS can be induced by a diverse range of causes including infection, neurogenic edema and trauma^39^. In neurogenic edema, damage to the central nervous system causes severe stress triggering ARDS^40^. Despite the differences between these origins of disease, they all result in damage to the lung epithelium that increases permeability, leading to pathological pulmonary edema and failure of the lung to undergo proper gas exchange^39^. In utilizing a model of ARDS that faithfully reproduces this pathology, efforts can be made to evaluate treatments that reduce or eliminate the damage caused by cellular injury. In other studies of EVLP and cytokine adsorption, the focus on extended cold ischemic storage has resulted in the conclusion that lung tissue may be perfused for longer but have not confronted a condition in which the lung is damaged to begin with. The similarity of LPS-induced ARDS to a clinical scenario of acute lung injury combined with the current context of an added cytokine adsorber would address the issue of ameliorating donor tissue damaged prior to storage.

In the pathology induced in this study, all donors developed mild to moderate ARDS with significantly lower gas exchange capacities as measured by the PaO_2_/FiO_2_ ratio before lung harvest. This adheres to the Berlin definition of the syndrome^34^ and the diagnosis of ARDS was further confirmed histologically. Pathologically, ARDS has previously been defined by diffuse alveolar damage in which hyaline membranes distinctively line alveolar spaces^41^. Edema and alveolar hemorrhage may be present as endothelial cells and pneumocytes undergo necrosis. In the model included in this study, mild hyaline membrane formation was observed in the tissue obtained following LPS administration. Further evidence of ARDS onset was supplemented by the blinded scoring conducted, which graded samples based on proteinaceous debris, thickening of the alveolar walls, hemorrhage, and atelectasis, and showed significant histological damage in LPS-treated lungs as compared to controls. The administration of LPS was also followed by a dramatic increase in early response cytokines, specifically IL-6, IL-8, IL-1β and TNF-α in all donors. ARDS has previously been reported to demonstrate an increase in these cytokines^10^. Proinflammatory cytokines TNF-α, IL–1β, IL-6, IL-8, and IL-18 have even been suggested as biomarkers of morbidity and mortality in ARDS^19^. TNF-α has been recognized in the pathogenesis of ARDS as a proinflammatory cytokine which acts downstream of pattern recognition receptors and IL-6 has been studied for its link to morbidity and mortality in ARDS^19,42^. In a discussion of potential subphenotypes of ARDS, a hyperinflammatory phenotype with high plasma levels of inflammatory biomarkers was found to have a different treatment response and correlate to higher mortality rates^43^.

Following the establishment of ARDS, harvested lungs were examined for recovery of function upon completion of cytokine adsorption during EVLP for four hours. While the PaO_2_/FiO_2_ ratios of non-treated lung did not reach acceptable levels for transplantation, treated ones had improved gas exchange capacity and most reached a PaO_2_/FiO_2_ ratio above 300. This is regarded as being clinically acceptable for transplantation^3^. Furthermore, lungs in the treated group experienced significantly reduced BALF levels of IL-1β relative to non-treated lungs. Other cytokines were also generally decreased throughout EVLP. This indicates a state of reduced inflammation when comparing the two conditions, further supported by the decreased number of immune cells and atelectasis seen on histological examination in the treated lungs relative to non-treated. The finding of decreased levels of IL-1β is of particular note given its previous identification as a prognostic indicator of nonrecovery during EVLP^44^. These results suggest restoration of lung function in the treated lungs.

The use of extracorporeal blood purification techniques to reduce tissue damage has been explored within the context of several surgical conditions associated with increased inflammatory cytokines^15,16,22^. Commercial products include cytokine adsorbers which remove substances through polymer beads. These devices target middle and low molecular weight molecules, thus reducing levels of diverse cytokines. They have so far been employed in vivo during human orthotopic heart transplantation and in human kidney transplantation settings^15,16,45^. In patients with severe sepsis and acute lung injury, the device was reported to have reduced the levels of IL-6, IL-8, IL-1β and TNF-α^21–24,46^. Based on the finding that the adsorption is beneficial in ameliorating healthy lungs damaged by ischemia, it was then utilized in this study for its potential to rescue ARDS donor lungs for LTx^26,27^.

To investigate the functionality of the adsorption in this setting, the transplanted lungs were followed for 48-hours and found to have a reduced need for inotropic support along with greater hemodynamic stability in both the one-step and two-step treatments. This mirrors the findings of studies of cytokine adsorption in septic patients, in whom the treatment reduced noradrenaline doses^18^. Furthermore, a randomized controlled trial in septicemia showed decreased inflammation by reducing IL-6 levels^21^. In this model, recipients were also found to have reduced cytokine levels and there were significant decreases in neutrophils and total white blood cells counts in the treated groups. Decreasing levels of cytokines is particularly important in ARDS given that clinical studies have shown increased IL-6 and TNF-α in the plasma and BALF samples of those who do not survive as well as a correlation of IL-6 with longer time spend on ventilation^11,19,47^. TNF-α produced from activated macrophages mediating the inflammatory response in ARDS will in turn activate neutrophils and along with other mediators, will lead to the recruitment of inflammatory cells to the alveoli^48^. Histologically, the treated lung tissue in this study showed a reduction in accumulated immune cells. This reinforces the finding that cytokine adsorption contributes to a decreased inflammatory state. Of note, the lung injury score for the one-step treatment fell between that of the two-step and non-treatment groups, a difference that was echoed in the TUNEL staining. This could indicate an additive effect on the part of the cytokine adsorber when administered both during EVLP and post-transplantation, rather than after transplantation alone.

As double LTx is not possible in pigs due to anatomical challenges on the right bronchus, a left LTx was conducted in this model. To evaluate the transplanted lung function, a right-sided pneumonectomy was consequently performed. Interestingly, there was no difference in the gas exchange capacity during the first day post transplantation between the groups. However, during the second day and especially after the right pneumonectomy, a significant difference in gas exchange could be seen between the two-step treatment and the non-treated, wherein the treated lungs performed better. The ratio in the one-step treatment in combination with the findings from the histological and apoptotic scores points toward a conclusion that the effects of the cytokine adsorber increase when used at two time points.

Differences in the response of grafts to transplantation between treatment groups pointed towards the wide scope of the cytokine adsorber’s effects. Post-transplantation, three recipients showed signs of septicemia. One recipient in the two-step treated group developed bacteremia 8 hours after transplantation but recovered with no subsequent signs. Another recipient in the non-treated group also developed septicemia post-transplantation which could not be overturned despite advanced intensive care and died 9 hours later. In the one-step treated group, one recipient developed mild septicemia with a severe tachyarrhythmia. The recipient was treated with albumin, a magnesium infusion, potassium, and intravenous lidocaine without any effect. The hemoadsorber was established and the tachyarrhythmia subsequently stabilized and resolved within 1 hour of hemoadsorption. The use of cytokine adsorption may have potentially mitigated the risk of developing fatal septicemia in the treated recipient. Additionally, the need for less inotropic support and greater hemodynamic stability of treated recipients could be attributed to reduced cytokine levels.

Interestingly, in one recipient in the two-step treated group, the graft developed dramatic pulmonary edema after 2 hours of EVLP. Up to 1.2 liters of fluid were drained from the trachea during EVLP and measures were taken during transplantation to fit the enlarged graft into the chest. By the end of the post-transplantation observational period, virtually all edema had been resorbed and the graft showed excellent gas exchange capacity and no signs of PGD, suggesting that the cytokine adsorption was of particular importance during hemoperfusion post-transplantation. Furthermore, the wet–dry ratios of the lung tissue when comparing the end of EVLP to the end of LTx showed a decrease between these time points. The wet–dry ratio assay was employed in order to determine the degree of pulmonary edema in the biopsies and provides evidence that the addition of a cytokine adsorber reduces accumulation of fluid in the tissue. These incidences of edema and septicemia illustrate how the addition of a cytokine adsorber may support restoration of non-acceptable donor lungs in the critical days immediately following transplantation.

The days following LTx are critical given the mortality associated with PGD, defined by PaO_2_/FiO_2_ ratios up to three days following surgery. In this study, after the addition of a cytokine adsorber in the first 12 hours after transplantation, five of six two-step treated recipients and two of four one-step treated recipients had no PGD at all. This contrasts with the five of six non-treated recipients who developed severe grade 3 PGD. Again, the two-step treatment appears to have an additive effect when compared to the rate of PGD in the one-step treatment group. This additive effect of treatment in both EVLP and post-transplant (the two-step group) with respect to to post-transplant alone (one-step group) is further emphasized in comparing the PaO_2_/FiO_2_ ratios at the end of the experiment (Fig. 5c). Not only are both groups improved relative to the non-treated recipients, but the two-step recipients are significantly higher than the one-step alone. Immune cell populations, particularly leukocytes, were significantly lower in the treated animals. This represents a curbed immunological response which could then facilitate the acceptance of a new organ during the initial post-transplantation period when the donor organ starts to be re-perfused. The diminished immunological response afforded by a cytokine adsorber could be responsible for the reduced incidence of PGD^49^.

There are limitations to the present study. One concern with cytokine adsorption in general is the potential for adsorption and removal of non-desired targets, with previous findings that plasma drug levels may decrease with treatment^27^. In this study, if the cytokine adsorption treatment groups had been experiencing reduced glucocorticoid and immunosuppressive agents, then higher rates of acute inflammation and signs of acute rejection would have been observed in the recipient during the clinical course and in the later histological examination. When translating the findings of this study to a clinical setting, concern over potentially diminishing drug levels should result in careful measurement of their plasma levels and precautions to maintain therapeutic levels. The intensive care required to sustain the pigs prohibits a longer follow-up over weeks or months, as might be desired to understand long-term outcomes, and thus the animals were only followed for 48 hours plus the time post-pulmectomy. This timeline did not allow for investigation of what effect cytokine adsorption may have on acute rejection or on CLAD. These conditions would be important to examine given the results found in the present study of reduced number of immune cell infiltration into the lung tissue. Two of the recipients in the one-step group developed postoperative pneumothorax. Chest x-ray showed that the lungs did not expand despite treatment with chest drainage. Because of underinflation of the lungs, higher PEEP was used to expand the lungs to facilitate contact with the chest wall and decrease air leakage. Subsequent expansion of the lung was confirmed with x-ray imaging. In consideration of the injury model used within this study, administration of LPS was chosen for its ability to reproduce an ARDS state, but it is limited as it does not represent a multi-factorial lung injury seen in human donor lungs. In addition, the study initially explored if there was any efficacy to the addition of cytokine adsorption at all and thus compared non-treatment to treatment both during EVLP and post-transplantation. Upon determination of a clinical and molecular improvement, an effort was made to refine when during this timeline cytokine adsorption held the greatest clinical utility and thus the one-step treatment group was added in. As a result, the addition of a third group presents some limitations to the data interpretation as differences may naturally arise with such a study design along with an inability to randomize the one-step treatment group as the other treatment and two-step groups had been randomized. Accordingly, because samples for cytokine analysis from the one-step group had to be analyzed at a different time than those from the non-treated and two-step groups, that data is presented separately within the supplement (Supplementary Fig. 1–4).

In summary, cytokine adsorption in the context of ARDS-injured lungs in this study has been shown to (i) reduce inflammation and restore pulmonary function during EVLP, (ii) restore function and decrease inflammation following transplantation, and (iii) reduce the incidence of PGD in transplanted recipients. The work outlined here represents the utilization of the cytokine adsorber in the context of lung transplantation using severely damaged donor lungs. It is thus envisioned that adsorption may be an intervention that could lead to the acceptance of more lungs for transplantation. It may also further increase the tolerability of such lungs in a recipient, a needed outcome given the role that PGD continues to play as the leading cause of early mortality and as a contributor to the development of chronic graft dysfunction.

## Methods

### Ethical considerations

The study was approved by the local Ethics Committee for Animal Research (Dnr 5.2.18-4903/16, and Dnr 5.2.18-8927/16) at Lund University. All animals received care according to the USA Principles of Laboratory Animal Care of the National Society for Medical Research, Guide for the Care and Use of Laboratory Animals, National Academies Press (1996). All animal handling, welfare monitoring, and euthanasia were attended to according to the guide for laboratory animals under the supervision of an on-site veterinarian.

### Animal Preparation

Thirty-two male and female adult farm-raised wild-type American Yorkshire pigs (*Sus scrofa domesticus*) were used in this study. Blood type was determined using Seraclone™ Anti-A (blood grouping reagent, Bio-Rad, Medical Diagnostics GmbH, Dreieich, Germany) prior to the experiment. Donor and recipient pairs were matched according to blood type and weight. The pairs were randomized prior to the start of the study to either the two- step treatment groups or the non-treated group. Given the findings of the two-step treatment group, the one-step treatment group was added to elucidate the differences between cytokine adsorption given during EVLP or post-transplantation.

Non-Treated: Lungs with LPS-induced ARDS receiving EVLP and LTx without cytokine adsorption (*n* = 6).

One-step Treated: Lungs with LPS-induced ARDS receiving EVLP without cytokine adsorption but with cytokine adsorption for 12 hours of extracorporeal cytokine hemoadsorption post-LTx (*n* = 4).

Two-step Treated: Lungs with LPS-induced ARDS receiving cytokine adsorption during EVLP and again for 12 hours of extracorporeal cytokine hemoadsorption post-LTx (*n* = 6).

A total of 32 pigs with a mean weight of 50 kg were premedicated with xylazine (Rompun® vet. 20 mg/mL; Bayer AG, Leverkusen, Germany; 2 mg/kg) and ketamine (Ketaminol® vet. 100 mg/mL; Farmaceutici Gellini S.p.A., Aprilia, Italy; 20 mg/kg). A peripheral intravenous (IV) line was inserted in the earlobe, and a urinary catheter was inserted in the bladder. General anaesthesia was accomplished with ketamine (Ketaminol® vet, Farmaceutici Gellini S.p.A.), midazolam (Midazolam Panpharma®, Panpharma Nordics AS, Oslo, Norway) and fentanyl (Leptanal®, Piramal Critical Care B.V., Lilly, France) infusions. Mechanical ventilation was established using a Siemens-Elema ventilator (Servo 900 C, Siemens, Solna, Sweden). The animals were intubated with a 7.5 size endotracheal tube. The ventilator was set to volume-controlled ventilation (VCV) with the flow pattern switch in “constant flow” which lowers the peak pressures according to the manufacturer’s instructions. Inspiration time is set to 25% with pause time 10% to give an I:E ratio of 1:2. Ventilation was adjusted to maintain carbon dioxide levels (PaCO_2_) between 33–41 mmHg. Tidal volume (Vt) was kept at 6–8 mL/kg. Dynamic compliance was calculated by the eq.${C}_{{dyn}}=\frac{{V}_{T}}{({peak\; pressure}\,-\,{PEEP})}$. A pulmonary artery catheter (Swan-Ganz CCOmbo V and Introflex, Edwards Lifesciences Services GmbH, Unterschleissheim, Germany) was inserted in the right internal jugular vein and an arterial line (Secalon-T^TM^, Merit Medical Ireland Ltd, Galway, Ireland) was placed in the right common carotid artery. 16 pigs were used as donor pigs and 16 pigs were used as recipients. Figure 1 shows the overview of the experimental set up. Dihydrostreptomycinsulfate (0.1 mL/kg, Boehringer Ingelheim Animal Health Nordics A/S, Copenhagen, Denmark) was given subcutaneously before initiation of surgery in all animals.

### Induction of ARDS using LPS in donor animals

Lipopolysaccharide (LPS) from Gram-negative bacteria *Escherichia coli* (O111:B4, Sigma-Aldrich, Merck KGaA, Darmstadt, Germany) was used to induce an ARDS according to the Berlin criteria^34^, as previously described^32^. LPS was diluted in saline solution and administered intravenously as an infusion (2 µg/kg/min) for one hour, after which point it was reduced by 50% for another hour. All LPS-treated animals developed hemodynamic instability and required continuous infusion of norepinephrine (40 µg/mL, 0.05–2 µg/kg/min, Pfizer AB, Sollentuna, Sweden) and dobutamine (2 mg/mL, 2.5–5 µg/kg/min, Hameln Pharma Plus GmbH, Hameln, Germany). Fluid loss was compensated with Ringer’s acetate (Baxter Medical AB, Kista, Sweden) in all animals.

The different ARDS stages were defined according to the Berlin definition^34^ using the PaO_2_/FiO_2_ ratio. Mild ARDS was defined as a ratio between 201 mmHg and 300 mmHg, moderate ARDS between 101 mmHg and 200 mmHg, and severe ARDS as ≤100 mmHg. Animals were confirmed as having ARDS following two separate arterial blood gases falling within the Berlin definition’s PaO_2_/FiO_2_ range within a 15-minute interval.

### Arterial blood gases

Arterial blood gases were analyzed every 30 min with an ABL 90 FLEX blood gas analyzer (Radiometer Medical ApS, Brønshøj, Denmark), and normalized to the blood temperature of 37 °C according to clinical standards in the donor animals and every hour during EVLP and after transplantation in the recipient animals.

### Hemodynamics

Hemodynamic parameters were measured every 30 min in the donor animals using thermodilution with a Swan-Ganz catheter and an arterial line. Heart rate (HR), systolic blood pressure (SBP), diastolic blood pressure (DBP), mean arterial pressure (MAP), central venous pressure (CVP), cardiac output (CO), systolic pulmonary pressure (SPP), diastolic pulmonary pressure (DPP), mean pulmonary pressure (MPP), pulmonary artery wedge pressure (PAWP), systemic vascular resistance (SVR), and pulmonary vascular resistance (PVR) were recorded.

### Pulmonary harvest after confirmed ARDS - donor

After confirmation of ARDS according to the Berlin definition with two blood gas values 15 min apart, a median sternotomy was performed. The pulmonary artery was cannulated via the right ventricle with a 28 F cannula secured by a purse string suture placed in the outflow tract of the pulmonary artery. A clamp was put on the superior vena cava, the inferior vena cava, and on the ascending aorta. The left atrium and inferior vena cava were opened. The lungs were perfused antegradely with 4 L of cold Perfadex® PLUS solution (XVIVO perfusion, Gothenburg, Sweden) distributed at a low perfusion pressure (<20 mmHg). The lungs were harvested *en bloc* in a standard fashion. The lungs were immersed in cold Perfadex® solution and put in cold storage at 4 °C for 2 hours.

### Ex vivo lung perfusion (EVLP)

EVLP was performed using Vivoline LS1 (XVIVO perfusion, Gothenburg, Sweden) on the harvested lungs *en bloc* and with a target perfusion of 40% of cardiac output, a tidal volume of 7 mL/kg body weight of the donor, respiratory rate (RR) of 7, 5 cmH_2_O PEEP for 4 hours^50,51^. The system was primed with Steen^TM^ Solution (XVIVO perfusion) and with red blood cells from the donor animal, drawn prior to LPS treatment, to reach a hematocrit level of 15–20% in the EVLP circuit. If the perfusate level dropped below 300 mL in the reservoir, additional Steen solution (XVIVO Perfusion) was added. EVLP physiology was recorded hourly during the 4-hour perfusion period. After 4 hours of EVLP the lungs were cooled down to 8–12 °C for approximately 45 min before transplantation.

### Cytokine adsorption during EVLP

During EVLP, in the two-step treated group, the perfusate was filtered continuously through an adsorbent filter (CytoSorb®, CytoSorbents Europe GmbH, Berlin, Germany) through a veno-venous shunt from the reservoir at a rate of 300 mL/min (Fig. 1b).

### Left lung transplantation - recipient

The lung transplantation was performed according to the protocol described by Mariscal et al.^52^.

In brief, the pulmonary hilum was dissected through a left thoracotomy and the left pulmonary artery, left atrium, and left bronchus were clamped individually. A left pneumonectomy of the native left lung was performed thereafter. The donor lung was sewn in, and the anastomosis of the bronchus was sutured using polydioxanone sutures (PDS 4-0, Ethicon, Somerville, NJ, USA). The atrial cuff and the pulmonary artery were sutured with polypropylene (Prolene 5-0, Ethicon) using a continuous pattern. All animals were immunosuppressed using tacrolimus (0.15 mg/kg, orally,Sandoz AS, Copenhagen, Denmark), and methylprednisolone sodium succinate (1 mg/kg, intravenously, Solumedrol, Pfizer). After suturing the bronchus, a bronchoscopy was done to confirm an open bronchial anastomosis.

### Extracorporeal hemoadsorption after transplantation

The two-step treated group that received cytokine adsorption during EVLP then received a further 12 hours of extracorporeal hemoadsorption also equipped with the cytokine adsorber following transplantation, while the one-step treated group did not receive any cytokine treatment during EVLP but received 12 hours of extracorporeal hemoadsorption also equipped with the cytokine adsorber following transplantation. This was accomplished through a veno-venous shunt using a hemodialysis catheter (Power-Trialysis® Slim-Cath™, Becton, Dickinson and Company, New Jersey, USA) inserted in the v. jugulars with a roller pump at a rate of 300 mL/min (Fig. 2c, video shown in the supplements).

### Recipient follow-up

The recipient animals were kept under anaesthesia using ketamine (Ketaminol® vet, Intervet AB, Stockholm, Sweden), midazolam (Midazolam Panpharma®, Panpharma Nordic, Oslo, Norway), fentanyl (Leptanal®, Piramal Critical Care B.V., Lilly, France), and rocuronium bromide (Esmeron®, Merck, Kenilworth, NJ, USA) infusions. 500 mg imipenem (Merck & Co. Inc., Kenilworth, NJ, USA) was given intravenously three times daily throughout the experimental timeframe. All animals (except for one that died of septicaemia 9 hours post-transplantation) were followed for at least 48 hours. Some animals, however, were followed up to 60–72 hours for logistical reasons. Dihydrostreptomycinsulfat (0.1 mL/kg, Boehringer Ingelheim Animal Health Nordics A/S) was given subcutaneously once daily. All animals were continuously immunosuppressed using tacrolimus (0.15 mg/kg, PO, (Sandoz AS), and methylprednisolone (1 mg/kg, intravenously, twice daily, Solumedrol, Pfizer). The ventilatory strategy followed for the recipients was to use as low pressures as possible in order to maintain adequate oxygenation and ventilation. This included maintaining a PEEP between 5–10 cmH_2_O and a peak pressure below 30 cmH_2_O.

### Right pneumonectomy

Given changes in positioning and preparation for the pneumonectomy, hemodynamic and blood gas measurements were taken immediately before the start of the procedure (labeled “Before Pneumonectomy” in Table 3). The pulmonary hilum was dissected through a mid-sternotomy, and a right pneumonectomy (including the accessory lobe) was performed to assess isolated function of the transplanted left lung. The recipient was followed for additional 4 hours following the right pneumonectomy before the experiment was terminated. During the 4 hours, the recipient was additionally monitored using a Swan-Ganz catheter as described in the Animal Preparation section. While the recipient was under one lung ventilation, the tidal volume and respiratory rate were adjusted to maintain a peak pressure less than 30 cmH_2_O.

### Measurements of cytokine levels in plasma and bronchoalveolar lavage fluid (BALF)

Measurements of cytokine levels in the plasma were taken at baseline, every 60 min in the donor animals, every hour during EVLP, and every 4th hour in the recipient animal. These levels were analyzed with the multiplex kit Cytokine & Chemokine 9-Plex Porcine ProcartaPlex™ Panel 1 (Thermo Fisher Cat. No. EPX090-60829-901, Thermo Fisher Scientific, Waltham, Massachusetts, US) according to the manufacturer’s instructions. The kit was run using a Bioplex-200 system (BioRad, Hercules, CA, USA). Nine cytokines were evaluated: IL-1β, IL-4, IL-6, IL-8, IL-10, IL-12p40, IFN-α, IFN-γ and TNF-α. Bronchoalveolar lavage fluid (BALF) was taken at baseline in donor animals, before lung harvest in donor animals, at the end of the EVLP, and at the end of the experiment.

### Blood cell counts

Leukocyte, neutrophils, and total white blood cell counts were measured using a Sysmex KX-21N automated haematology analyser (Sysmex, Milton Keynes, UK) every 30 min in the donor animals, every hour during EVLP, and every 1–6th hour after LTx. Blood samples anti-coagulated with EDTA were kept at 4 °C until analysis.

### Histopathological Analyses

Baseline lung biopsies were taken from the right lobe before LPS administration through a right thoracotomy. Biopsies were also taken from the right lower lobe just before lung harvest after confirmed ARDS. When the lung was connected to EVLP, biopsies were taken from the right lower lobe at its initiation and then every hour throughout EVLP. Biopsies were also taken from the transplanted left lung after completion of the experiment. Biopsies were fixed in 10% neutral buffered formalin solution (Sigma Aldrich, Germany) at 4 °C overnight. Formalin-fixed tissues were subjected to a graded ethanol series and isopropanol (both Fisher Scientific) prior to paraffin embedding (Histolab Products AB, Gothenburg, Sweden). 4 μm sections were cut and after de-paraffinization, the sections were stained with hematoxylin and eosin (Merck Millipore, Germany) followed by dehydration in consecutively graded ethanol and xylene solutions. Dried sections were mounted with Pertex (Histolab). Brightfield images were acquired with a Nikon Eclipse Ts2R microscope (Nikon, Tokyo, Japan). Histology of all donor lungs across time points are presented in the following figures: Supplementary Fig. 8 for non-treated, Supplementary Fig. 9 for one-step treated and Supplementary Fig. 10 for two-step treated lungs, respectively.

Images from each animal were scored independently for lung injury by three blinded scorers with experience in porcine lung injury models on the basis of number of inflammatory cells, presence of hyaline membranes, level of proteinaceous debris, thickening of the alveolar wall, enhanced injury, hemorrhage and atelectasis using a modification of previously described scoring methodology^53^. Scores were given on a scale of 0 to 8 for each feature and reported as average of the sum of the characteristic scores.

Tissue sections were then also assessed for late apoptosis via TdT-mediated dUTP nick end-labelling (TUNEL) assay, which was performed according to the manufacturer’s instructions (Abcam catalog #ab206386, Abcam, Cambridge, UK). Samples were randomly selected from each group, with five slides each from the baseline and confirmed ARDS groups, as well as 6 slides from the EVLP groups. All slides from the end of observation of transplanted recipients were stained. Slide images were then acquired using brightfield with a 40 × objective with two lung pieces scanned per slide with an Olympus VS-120-S6 virtual slide scanner (Olympus, Hamburg, Germany). TUNEL positive cell counts per piece were determined and normalized to the lung tissue area represented in the TUNEL score using Fiji ImageJ 1.53 M software^54^.

### Wet dry-weight ratio

Pulmonary edema was examined by measuring the wet weight to dry weight ratio in lung tissue from the lower lobe after EVLP in the left lung and after 48 hours of transplantation in the left (transplanted) lung. Proximal lung tissue pieces harvested were weighed, lyophilized for 24 hours, and then weighed again. The ratio between the wet and dry weight was then calculated.

### Primary graft dysfunction staging

The primary graft dysfunction (PGD) was staged according to the Pa0_2_/FiO_2_ ratio using the ISHLT guidelines^55^. Presence of lung infiltrate to fulfill the criteria of PGD was assessed through imaging conducted with a mobile C-arm x-ray machine (Siemens, Munich, Germany).

### Calculations and statistics

Data collection was conducted with Microsoft Excel Version 16.46 and GraphPad Software Version 9. Image analysis for TUNEL staining was also conducted with Fiji ImageJ 1.53 M. Continuous variables were reported as mean and standard deviation (SD). Statistically significant differences between groups were tested with the Student’s *T*-test when comparing two groups and within groups with ANOVA when data were normally distributed. Most analyses were conducted with the Mann–Whitney test and the Kruskal–Wallis tests as data were not normally distributed. A Chi-Squared test was performed to analyze observed frequencies of categorical variables. All statistical analysis was performed using GraphPad Prism 9.1. Significance was defined as: *p* < 0.001 (***), *p* < 0.01 (**), *p* < 0.05 (*), and *p* > 0.05 (not significant).

### Reporting summary

Further information on research design is available in the Nature Research Reporting Summary linked to this article.

## Supplementary information

Supplementary Information

Reporting Summary

Description of Additional Supplementary Files

Supplementary Video 1

## Data Availability

The authors declare that all data supporting the findings are available within in the paper and Supplementary Information and from the corresponding author upon reasonable request. The source data underlying Fig. 2a-d, n; 3a–f, l; Fig. 4a–d; 5a, b are provided in the Source Data file. Source data are provided with this paper.

## Source data

Source Data
