# Supplementary material for: Reduction of primary graft dysfunction using cytokine adsorption during organ preservation and after lung transplantation
Source: Nat Commun. 2022 Jul 26;13:4173. doi: 10.1038/s41467-022-31811-5 (PMC9325745; doi:10.1038/s41467-022-31811-5)
Supplement: Supplementary file 1 — Supplementary Information [file 41467_2022_31811_MOESM1_ESM.docx]

**Reduction of primary graft dysfunction using cytokine adsorption during organ preservation and after lung transplantation**

Haider Ghaidan MD^1,2,3,4^, Martin Stenlo MD, PhD^2,3,4,5^, Anna Niroomand BA^2,3,4,6^, Margareta Mittendorfer BSc MSc^2,3,4^, Gabriel Hirdman MD^2,3,4^, Nika Gvazava MD^2,4,7^, Dag Edström MD^2,3,4,5^, Iran A. N. Silva MSc^2,4,7^, Ellen Broberg MD, PhD^2,3,4,5^, Oskar Hallgren, PhD^2,3,4^, Franziska Olm PhD^2,3,4^, Darcy E Wagner, PhD^2,4,7^, Leif Pierre PhD^1,2,3,4^, Snejana Hyllén MD, PhD^2,3,4,5^, Sandra Lindstedt MD, PhD^1,2,3,4^

^1^Dept. of Cardiothoracic Surgery and Transplantation, Skåne University Hospital, Lund, Sweden.^2^Wallenberg Center for Molecular Medicine, Lund University, Sweden. ^3^Dept. of Clinical Sciences, Lund University, Sweden. ^4^ Lund Stem Cell Center, Lund University, Sweden. ^5^Dept. of Cardiothoracic Anaesthesia and Intensive Care, Skåne University Hospital, Lund, Sweden. ^6^Rutgers Robert University, New Brunswick, New Jersey, USA. ^7^Dept. of Experimental Medical Sciences, Lung Bioengineering and Regeneration, Lund University, Sweden.

*Address for reprint requests and other correspondence*: S. Lindstedt, Department of Cardiothoracic Surgery and Transplantation, Skåne University Hospital, Lund, SE-221 85 Lund, Sweden (email: [sandra.lindstedt_ingemansson@med.lu.se](mailto:sandra.lindstedt_ingemansson@med.lu.se))

**Supplementary Information**

Table of Contents

[**Supplementary Figures** 3](#_Toc103449530)

[**Supplementary Figure 1: Establishment of Acute Respiratory Distress Syndrome (ARDS) lung injury in the donor.**. 3](#_Toc103449531)

[**Supplementary Figure 2: Cytokine levels in plasma for the later one-step treated group.** 4](#_Toc103449532)

[**Supplementary Figure 3:** **Plasma cytokine levels for the one-step treated group.** 4](#_Toc103449533)

[**Supplementary figure 4: Cytokines levels in bronchoalveolar lavage fluid (BALF) through the course of the experiment for the one-step treated group.**. 5](#_Toc103449534)

[**Supplementary Figure 5:** **Histological evaluation at baseline and following lipopolysaccharide (LPS) from biopsies from the right lower lungs representative of n=16 lungs.** 6](#_Toc103449535)

[**Supplementary Figure 6: Histology following four hours of ex vivo lung perfusion (EVLP) from biopsies taken from the right lower lungs representative of n=16 lungs.** 7](#_Toc103449536)

[**Supplementary Figure 7: Histology following lung transplantation (LTx) from biopsies taken from the left lower lung representative of n=16 lungs.** 8](#_Toc103449537)

[**Supplementary Figure 8: Donor lung histology over the course of the experiment for individual donor/recipient pairs in the non-treated group representative of n=16 lungs.**. 9](#_Toc103449538)

[**Supplementary Figure 9: Donor lung histology over the course of the experiment for individual donor/recipient pairs in the one-step treated group representative of n=16 lungs.** 10](#_Toc103449539)

[**Supplementary Figure 10: Donor lung histology over the course of the experiment for individual donor/recipient pairs in the two-step treated group representative of n=16 lungs.**. 11](#_Toc103449540)

[**Supplementary Figure 11: Evaluation of late apoptosis in lung tissue at baseline and following lipopolysaccharide (LPS).** 12](#_Toc103449541)

[**Supplementary Figure 12. Evaluation of late apoptosis in lung tissue following four hours of ex vivo lung perfusion (EVLP) representative of n=16 lungs.** 12](#_Toc103449542)

[**Supplementary Figure 13. Evaluation of late apoptosis in lung tissue following the end of observation in recipients (LTx end) representative of n=16 lungs.** 13](#_Toc103449543)

[**Supplementary Figure 14. Pulmonary gas exchange and peripheral differential blood cell counts during ex vivo lung perfusion (EVLP).** 14](#_Toc103449544)

[**Supplementary Figure 15. Wet/dry ratios of lung biopsy tissue.** 15](#_Toc103449545)

[**Supplementary Videos** 15](#_Toc103449546)

[**Supplementary video 1. Setup of cytokine adsorption system using extracorporeal hemoperfusion in the post-transplantation recipient**. 15](#_Toc103449547)

[**Supplementary Tables** 15](#_Toc103449548)

[**Supplementary Table 1. Physiological status of donors treated with lipopolysaccharide (LPS) over time until confirmation of acute respiratory distress syndrome (ARDS).** 15](#_Toc103449549)

[**Supplementary Table 2.** **Physiological status of recipients with or without treatment post right pneumonectomy.** 17](#_Toc103449550)

[**Supplementary Table 3. Overview of exact p-values present in figure 2-5.** 18](#_Toc103449551)

**Supplementary Figures**


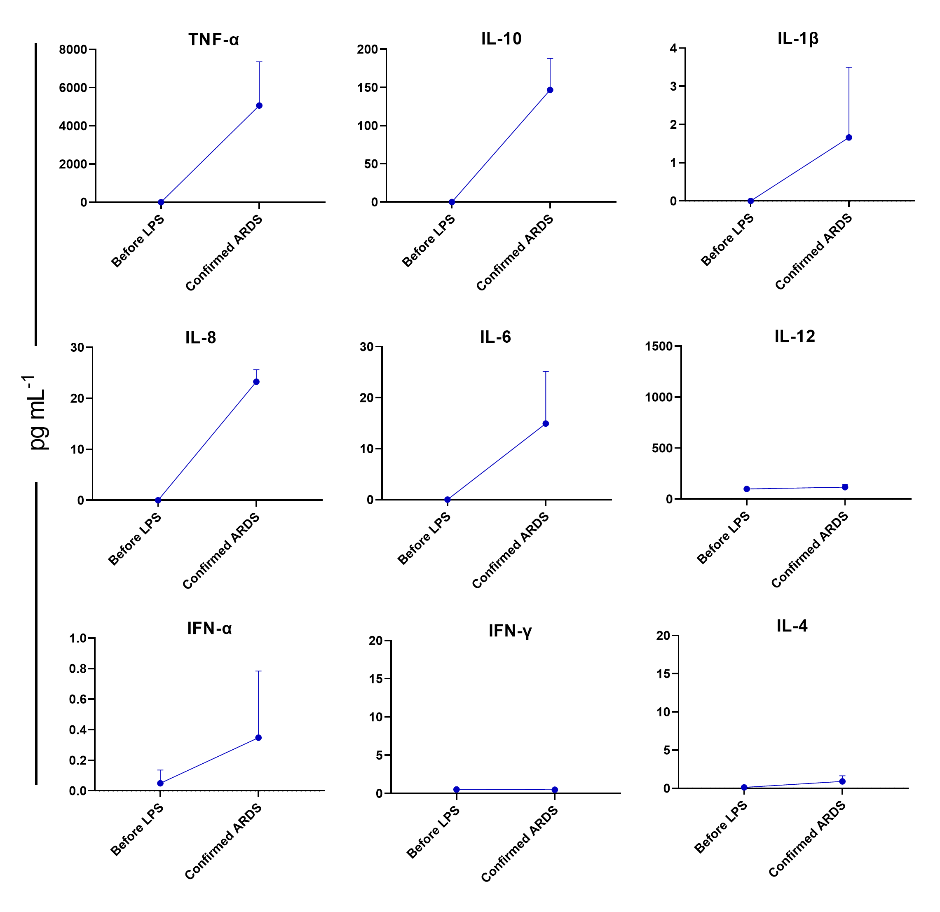


**Supplementary Figure 1: Establishment of Acute Respiratory Distress Syndrome (ARDS) lung injury in the donor.** Cytokine levels in plasma for later one-step treated group at baseline and time of confirmed ARDS (n=4). All values represent the mean ± standard deviation.


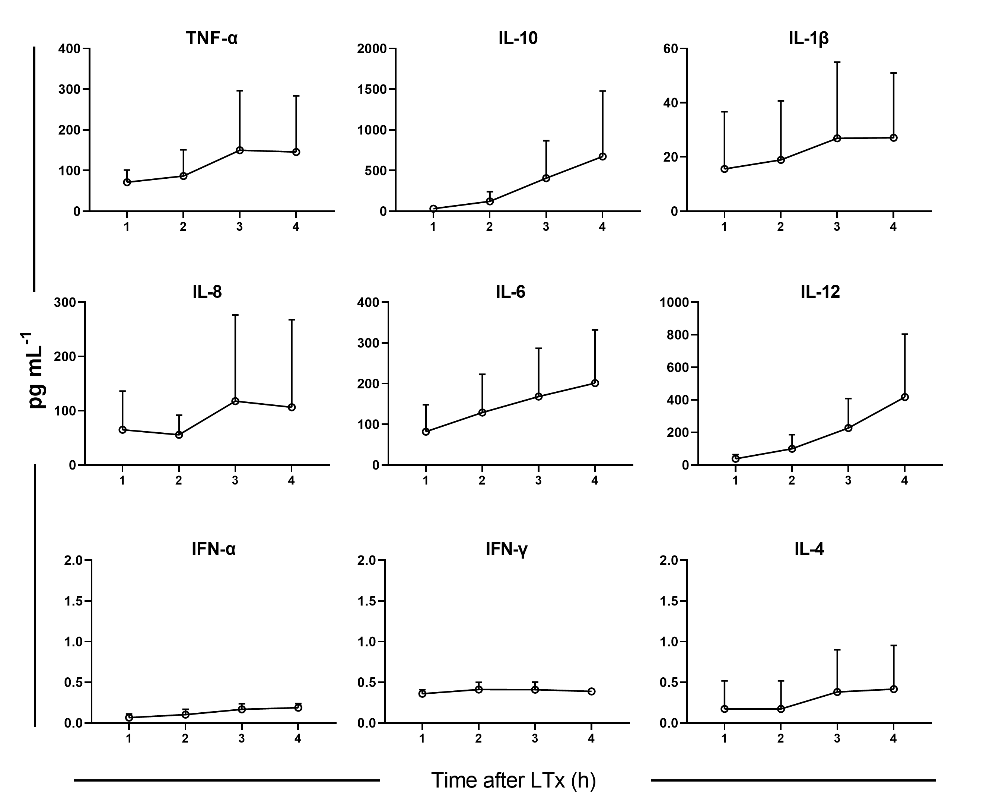


**Supplementary Figure 2: Cytokine levels in plasma for the later one-step treated group.** Levels were measured every hour during the 4h EVLP time period (n=4). All values represent the mean ± standard deviation.


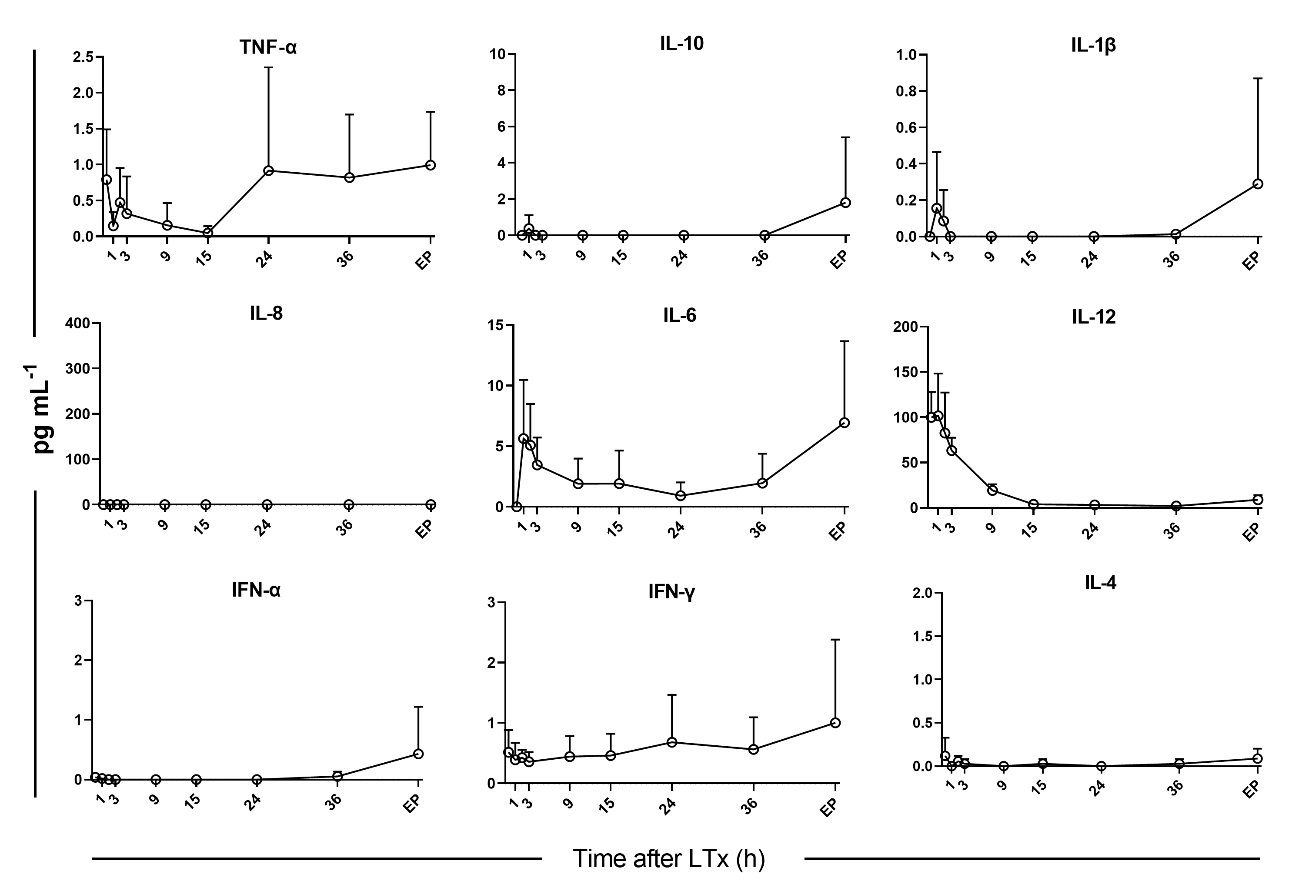


**Supplementary Figure 3:** **Plasma cytokine levels for the one-step treated group.** Levels were monitored throughout the 48-hour period following transplantation, with 1 hour marking the time elapsed since the start of treatment. EP stands for endpoint of the experimental timeline (n=4). All values represent the mean ± standard deviation.


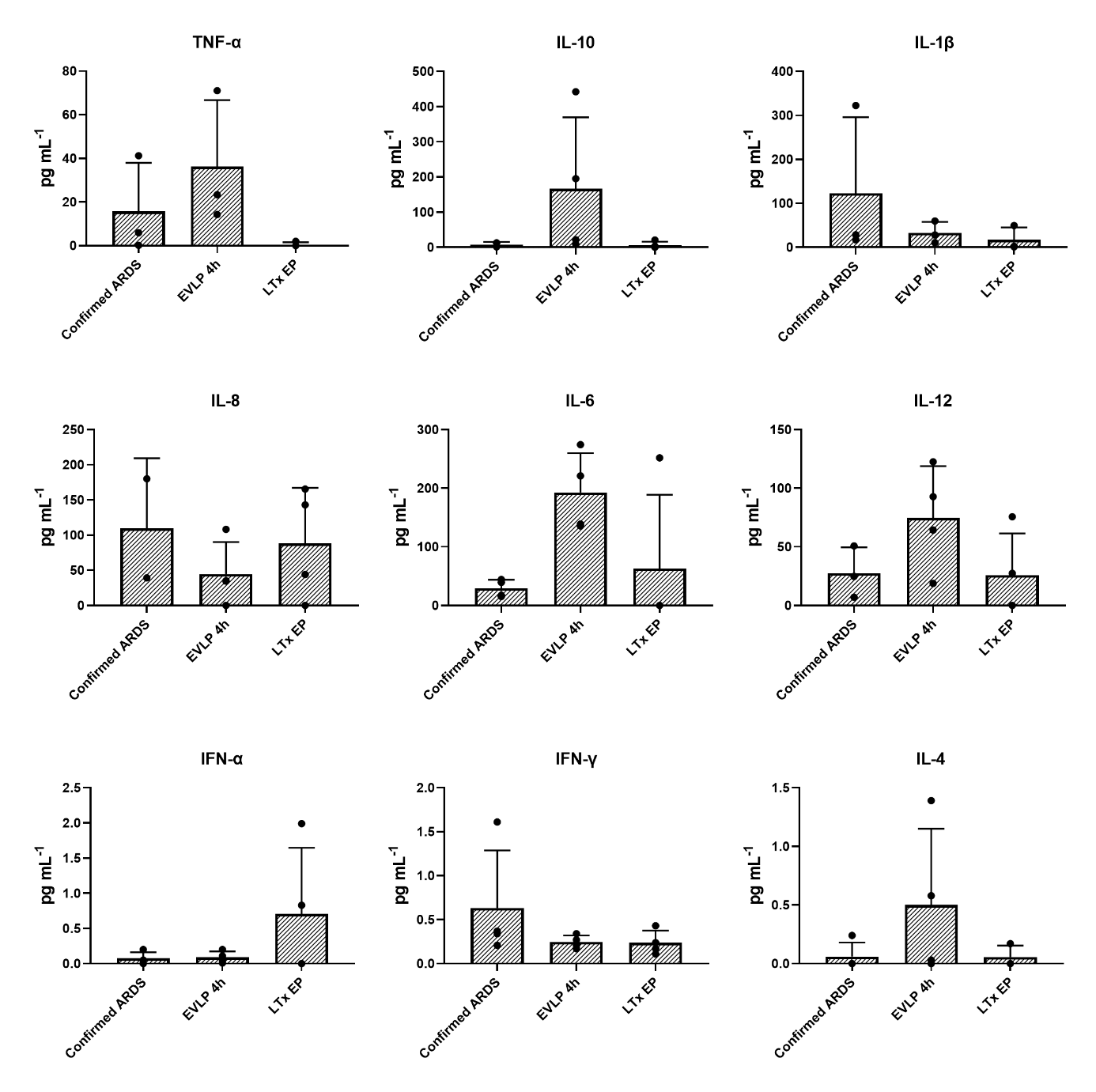
**Supplementary figure 4: Cytokines levels in bronchoalveolar lavage fluid (BALF) through the course of the experiment for the one-step treated group.** BALF was tested for cytokine concentrations at the point of confirmed acute respiratory distress syndrome (ARDS), the end of the ex vivo lung perfusion (EVLP) treatment and at the termination of the experiment (LTx EP, n=4). All values represent the mean ± standard deviation.

**
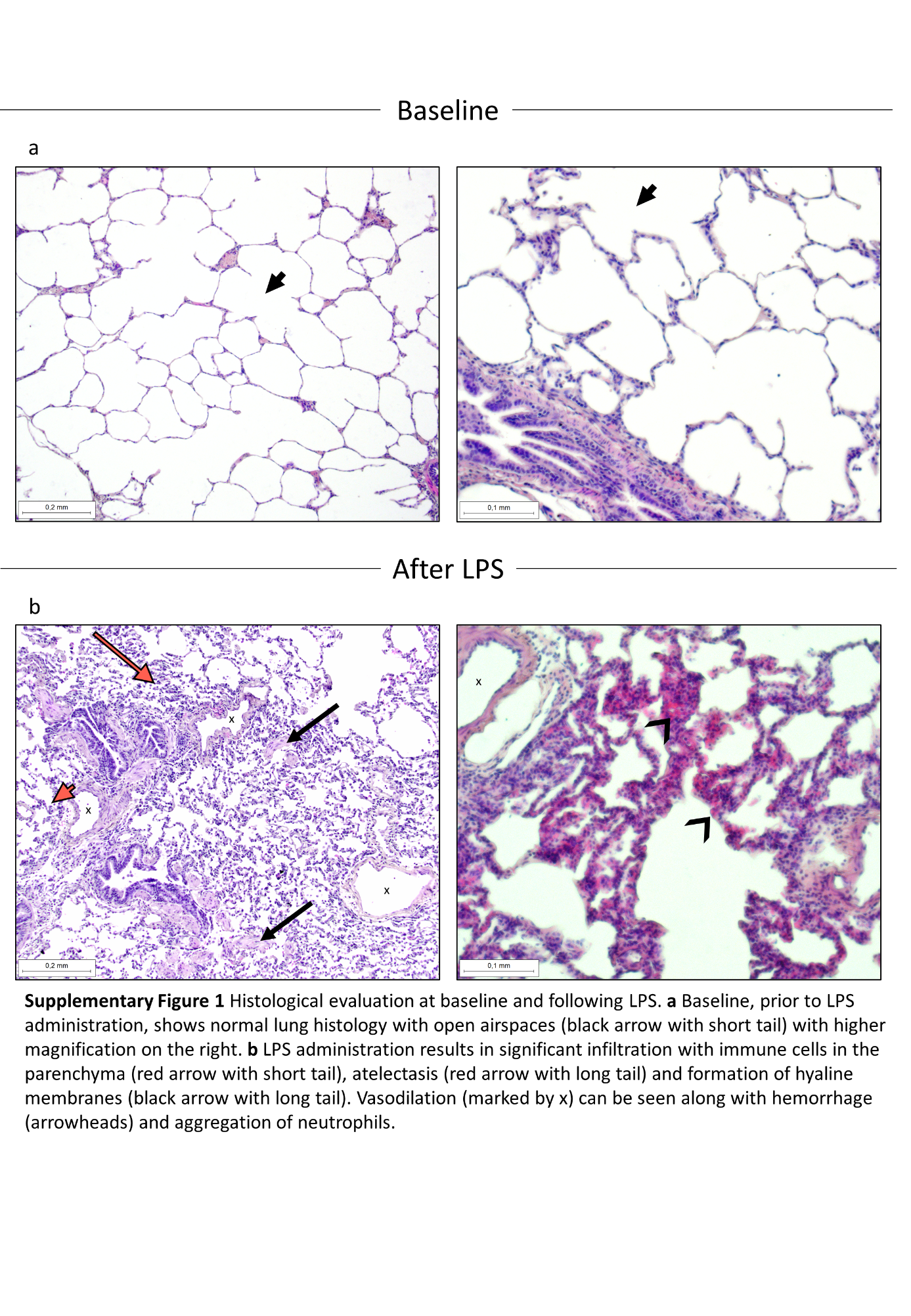
**

**Supplementary Figure 5:** **Histological evaluation at baseline and following lipopolysaccharide (LPS) from biopsies from the right lower lungs representative of n=16 lungs.** **a** Baseline, prior to LPS administration, shows normal lung histology with open airspaces (black arrow with short tail). **b** LPS administration results in significant infiltration with immune cells in the parenchyma (red arrow with short tail), atelectasis (red arrow with long tail) and formation of hyaline membranes (black arrow with long tail). Vasodilation (marked by x) can be seen along with hemorrhage (arrowheads) and aggregation of neutrophils. Scale bars represent 0.2 mm (left) and 0.1 mm (right), respectively.

**
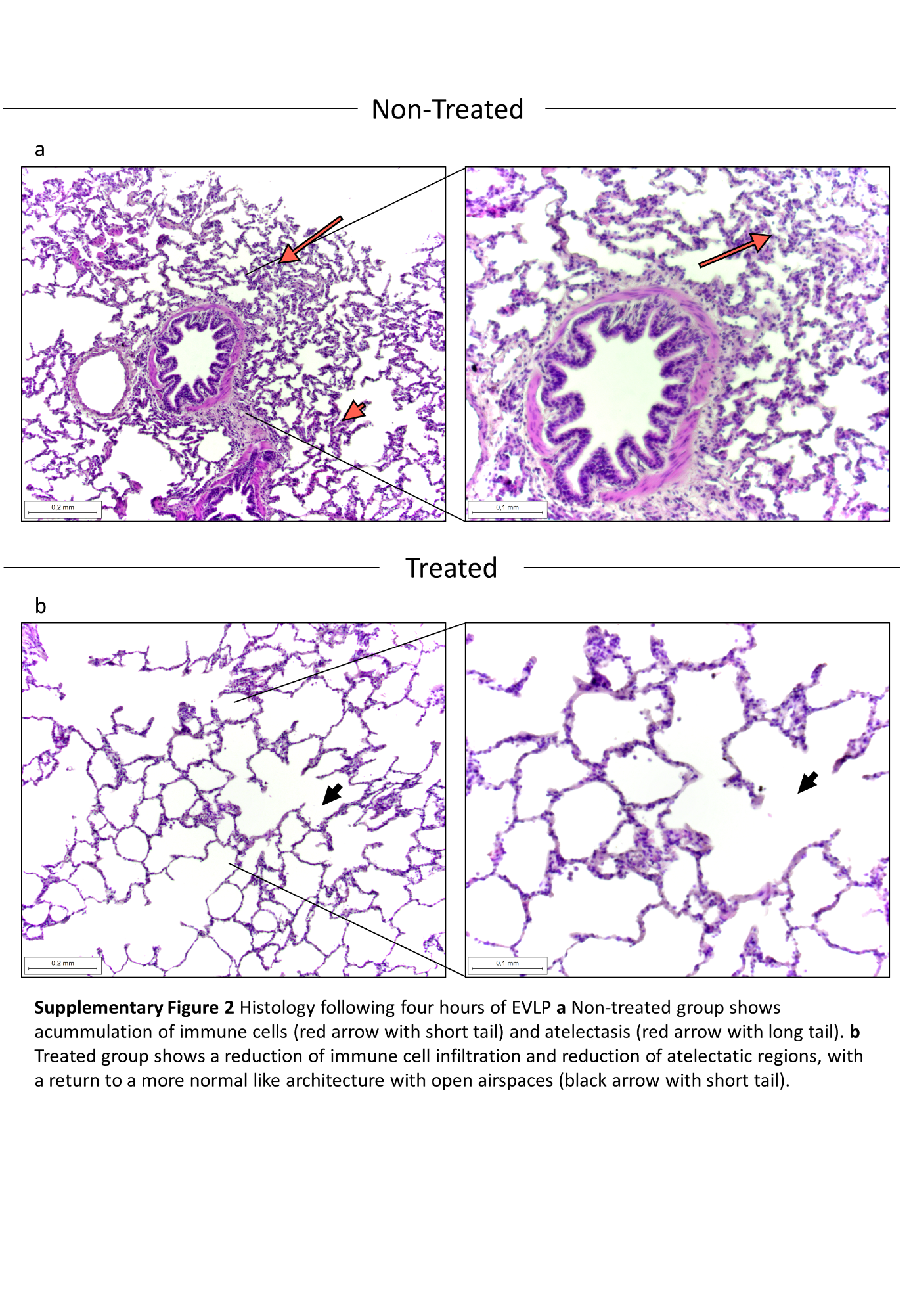
**

**Supplementary Figure 6: Histology following four hours of ex vivo lung perfusion (EVLP) from biopsies taken from the right lower lungs representative of n=16 lungs.** **a** Non-treated group shows accumulation of immune cells (red arrow with short tail) and atelectasis (red arrow with long tail). Non-treated group also includes the one-step in this instance as they did not receive cytokine adsorption during EVLP. **b** Treated group shows a reduction of immune cell infiltration and reduction of atelectatic regions, with a return to a more normal like architecture with open airspaces (black arrow with short tail). Scale bars represent 0.2 mm (left) and 0.1 mm (right), respectively.

**
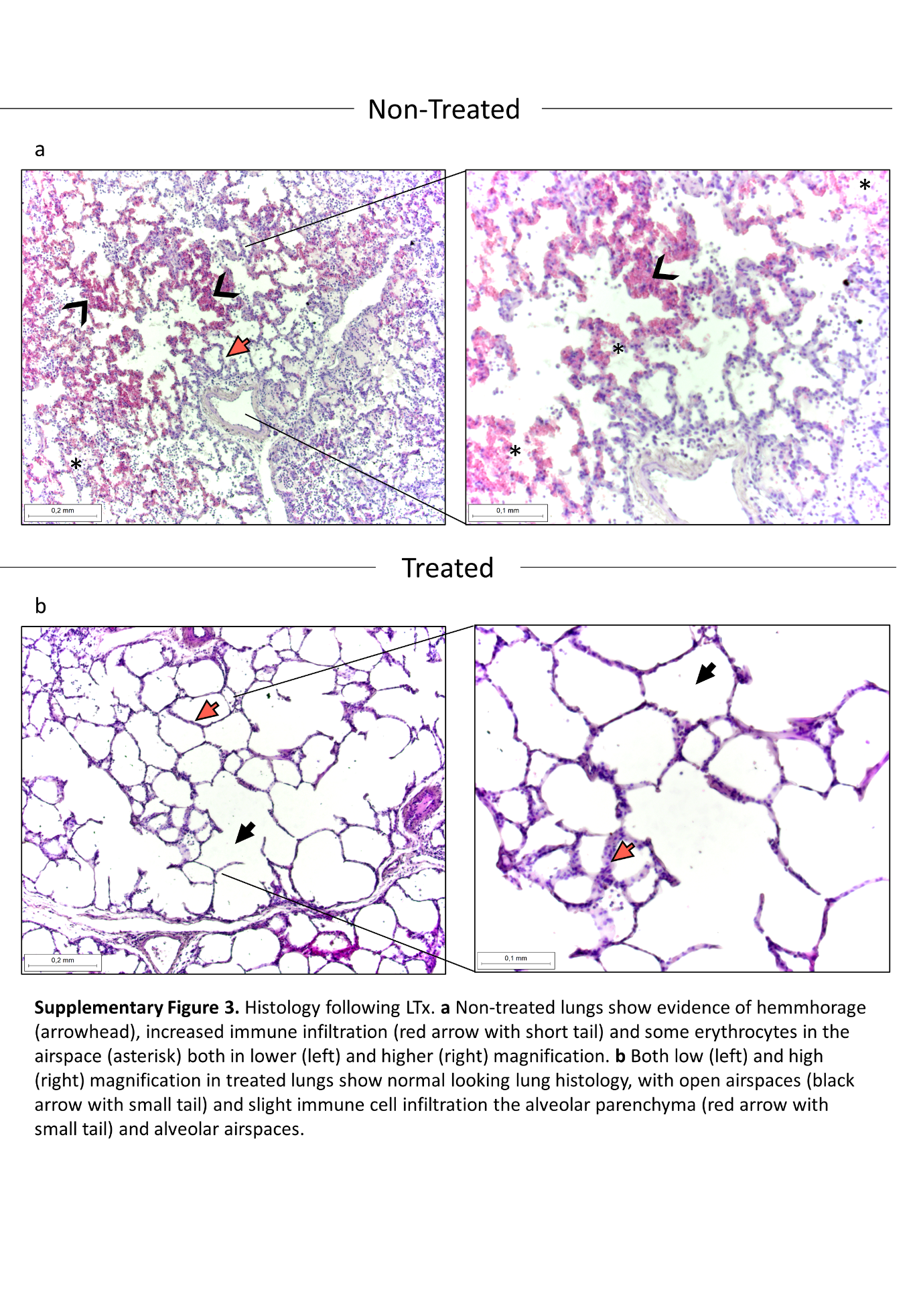
**

**Supplementary Figure 7: Histology following lung transplantation (LTx) from biopsies taken from the left lower lung representative of n=16 lungs.** **a** Non-treated lungs show evidence of hemorrhage (arrowhead), increased immune infiltration (red arrow with short tail) and some erythrocytes in the airspace (asterisk) both in lower (left) and higher (right) magnification. **b** Both low (left) and high (right) magnification in treated lungs (one-step and two-step groups) show normal looking lung histology, with open airspaces (black arrow with small tail) and slight immune cell infiltration the alveolar parenchyma (red arrow with small tail) and alveolar airspaces. Scale bars represent 0.2 mm (left) and 0.1 mm (right), respectively.


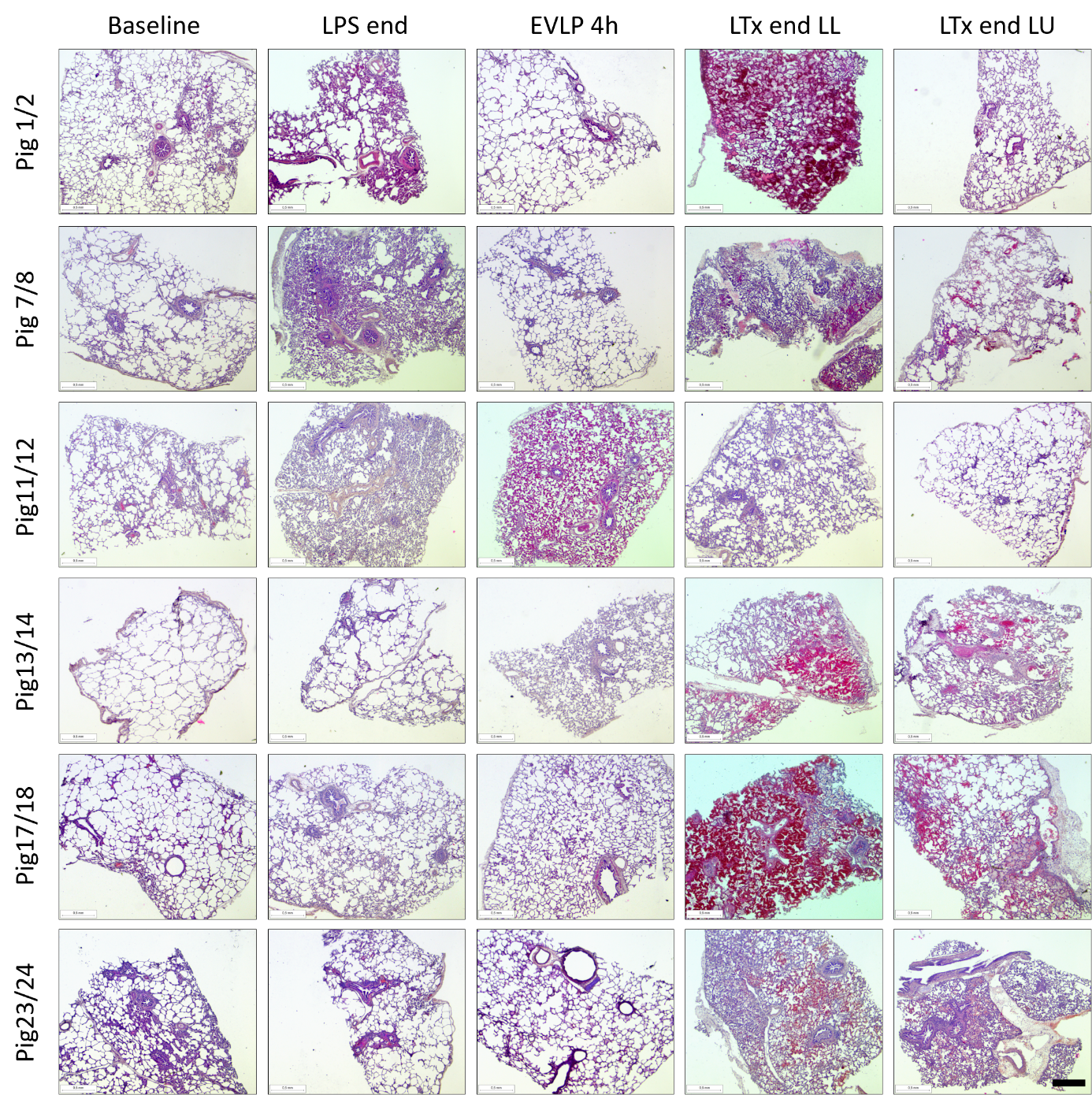


**Supplementary Figure 8: Donor lung histology over the course of the experiment for individual donor/recipient pairs in the non-treated group representative of n=16 lungs.** Histology of each non-treated donor lung across timepoints including baseline, confirmed acute respiratory distress syndrome (ARDS) referred to as LPS end, after 4 hours of ex vivo lung perfusion (EVLP) and after lung transplantation (LTX end) with samples from both the left lower lobe (LL) and left upper lobe (LU)). Scale bar represents 500 µm.


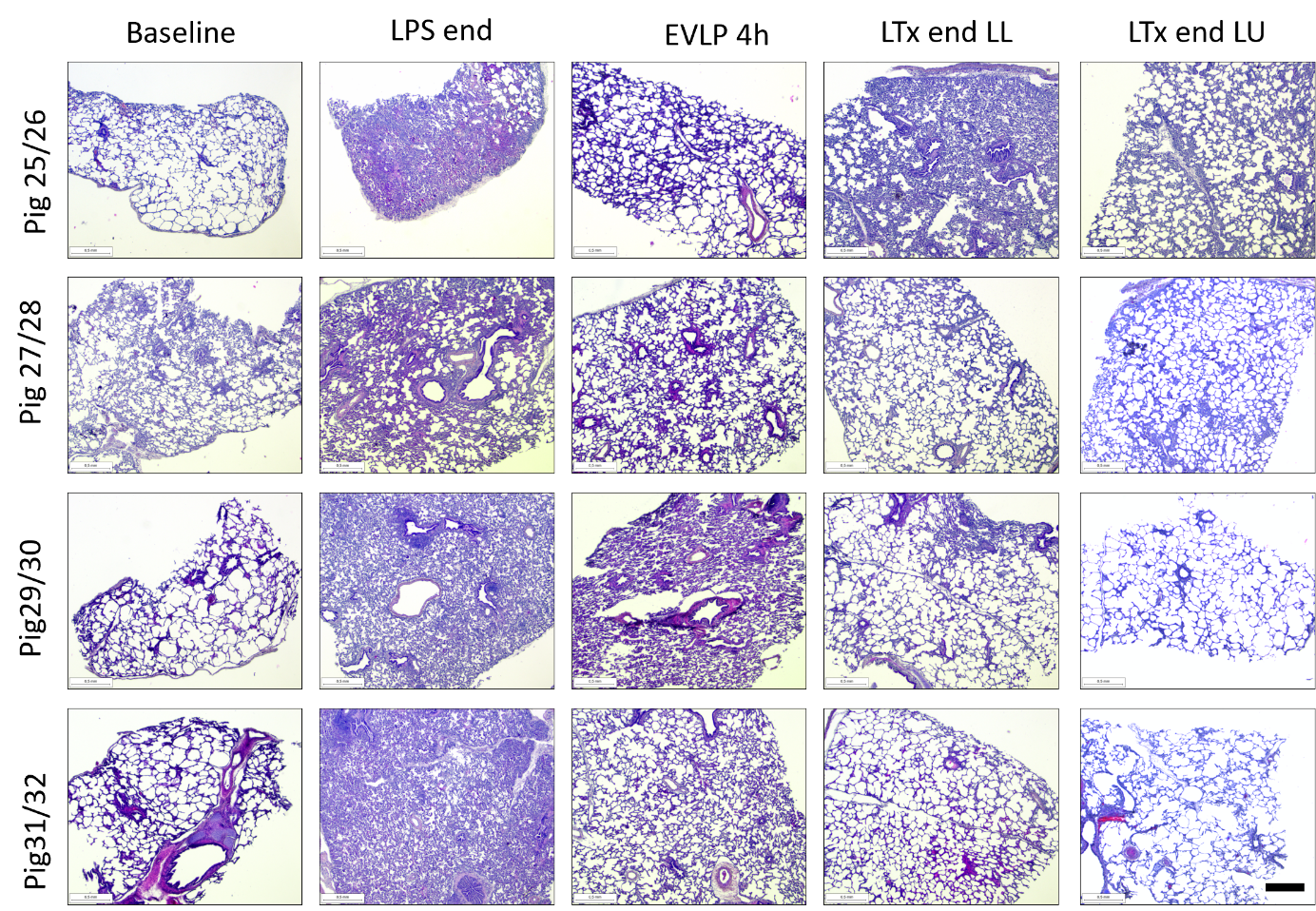


**Supplementary Figure 9: Donor lung histology over the course of the experiment for individual donor/recipient pairs in the one-step treated group representative of n=16 lungs.** Histology of each one-step treated donor lung across timepoints including baseline, confirmed acute respiratory distress syndrome (ARDS) referred to as LPS end, after 4 hours of ex vivo lung perfusion (EVLP) and after transplant (LTX end) with samples from both the left lower lobe (LL) and left upper lobe (LU)). Scale bar represents 500 µm.

**
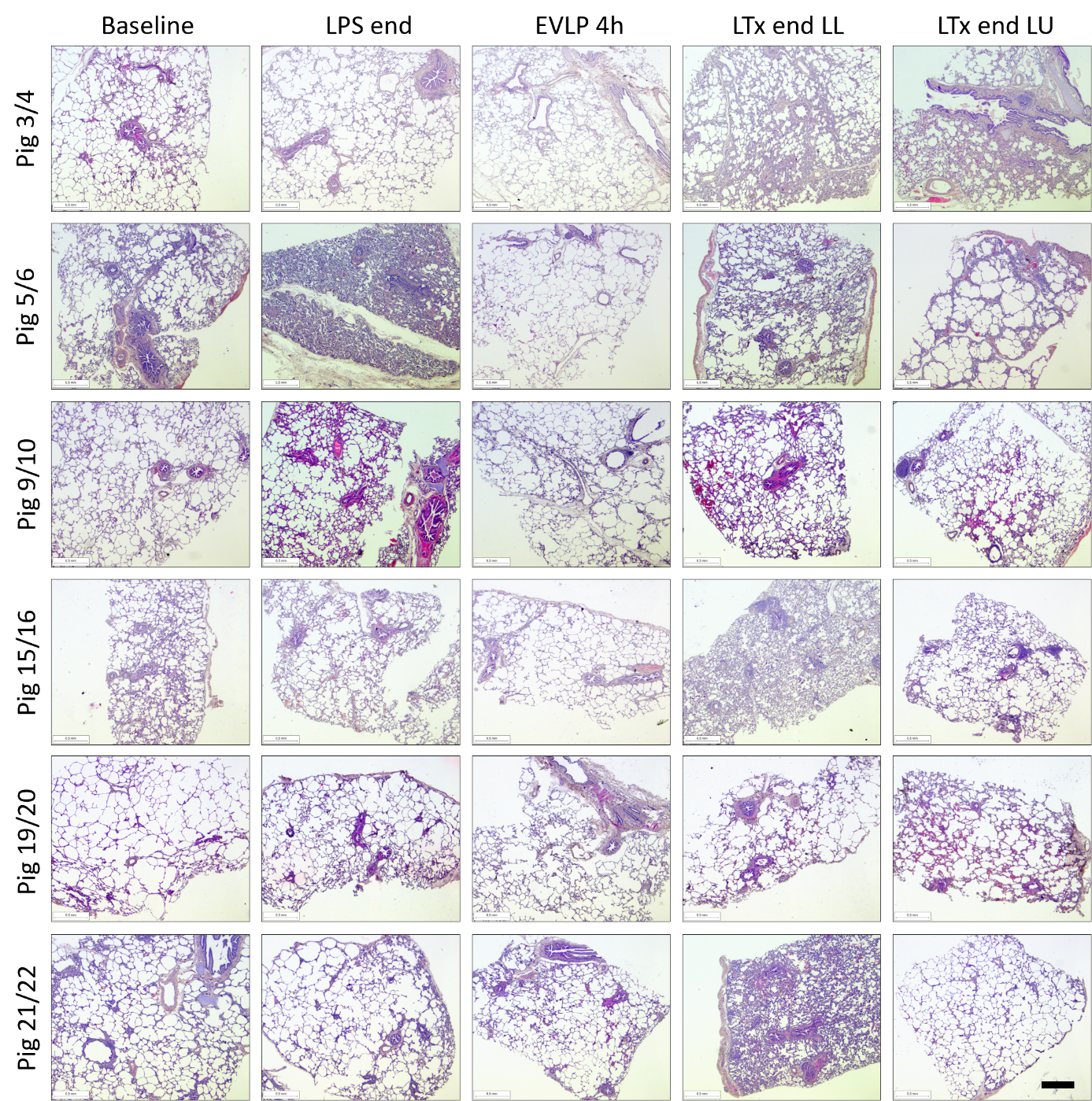
**

**Supplementary Figure 10: Donor lung histology over the course of the experiment for individual donor/recipient pairs in the two-step treated group representative of n=16 lungs.** Histology of each two-step treated donor lung across timepoints including baseline, confirmed acute respiratory distress syndrome (ARDS) referred to as LPS end, after 4 hours of ex vivo lung perfusion (EVLP) and after transplant (LTX end) with samples from both the left lower lobe (LL) and left upper lobe (LU)). Scale bar represents 500 µm.

**
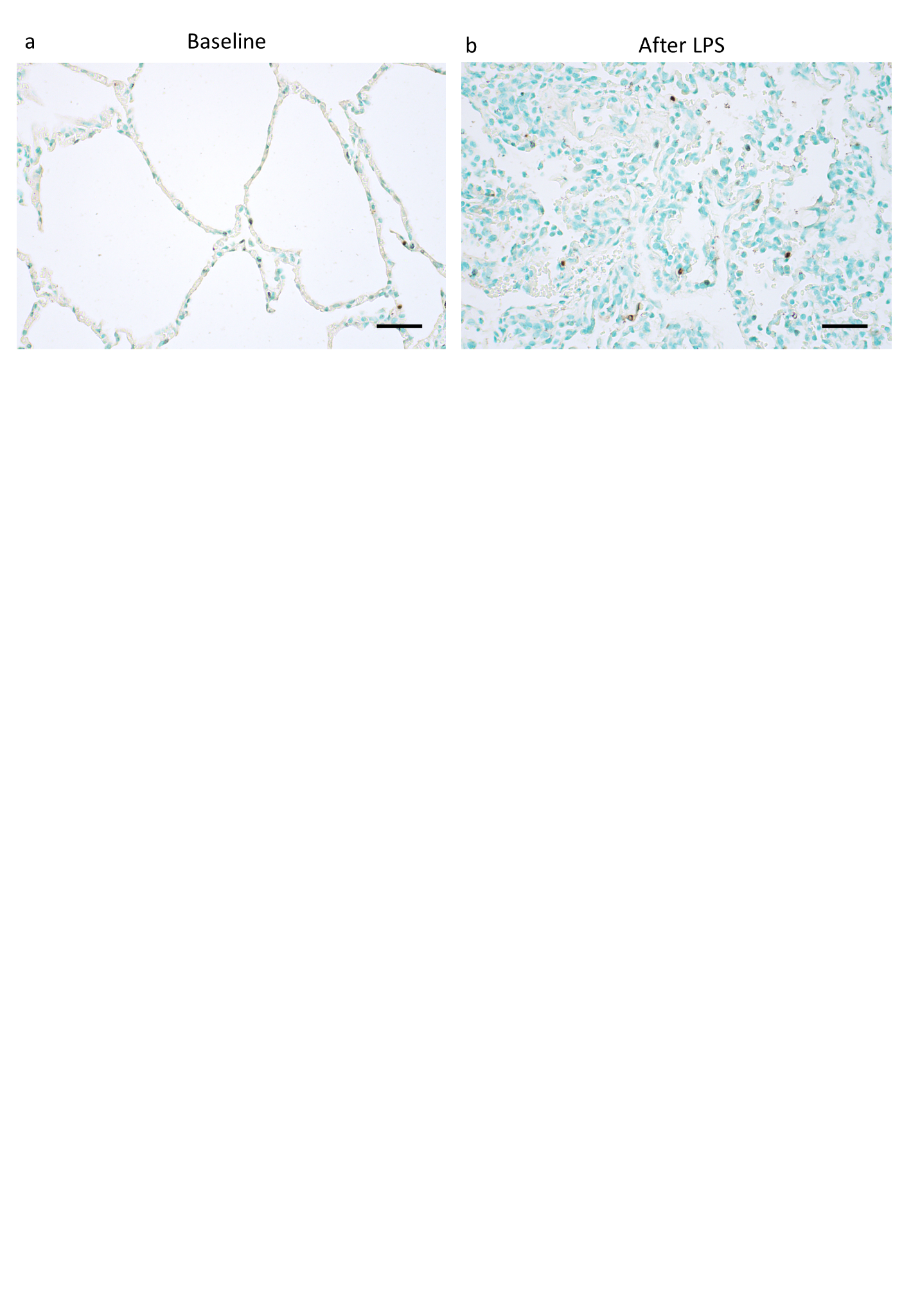
Supplementary Figure 11: Evaluation of late apoptosis in lung tissue at baseline and following lipopolysaccharide (LPS).** Bright field images of terminal deoxynucleotidyl transferase dUTP nick end labeling (TUNEL) staining (brown) as marker for late apoptosis with methyl green as counter stain (left images). **a** Baseline, prior to lipopolysaccharide (LPS) administration, shows normal lung morphology with open airspaces and very low levels of apoptotic cells. **b** LPS administration results in increased levels of apoptosis. Scale bars represent 50 µm.

**
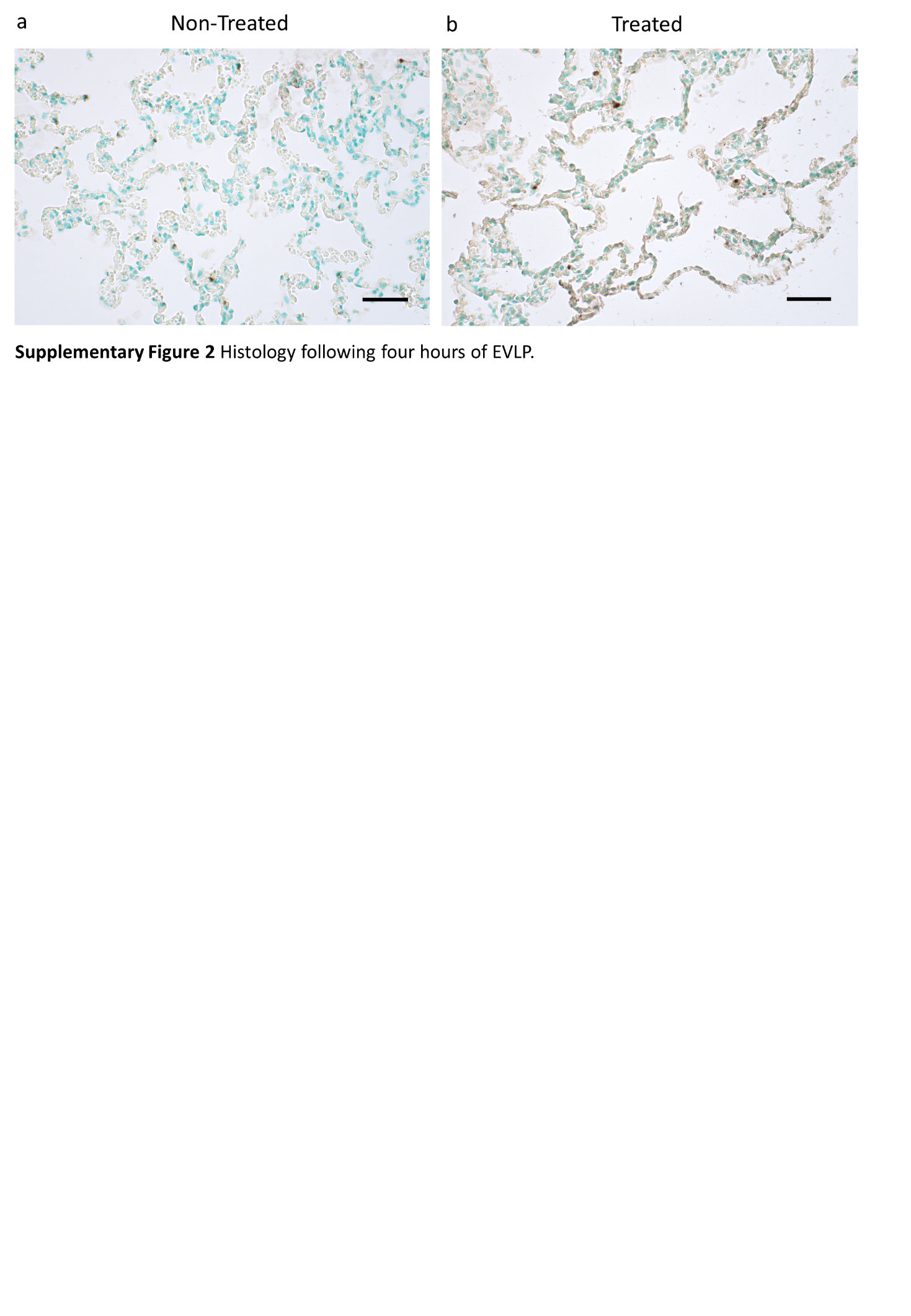
**

**Supplementary Figure 12. Evaluation of late apoptosis in lung tissue following four hours of ex vivo lung perfusion (EVLP) representative of n=16 lungs.** Bright field images of terminal deoxynucleotidyl transferase dUTP nick end labeling (TUNEL) staining (brown) as marker for late apoptosis with methyl green as counter stain (left images). Both **a** and **b** demonstrate representative images of the non-treated and treated groups on the left and right respectively, with signs of increased levels of apoptotic cells compared to baseline. Scale bars represent 50 µm.

**
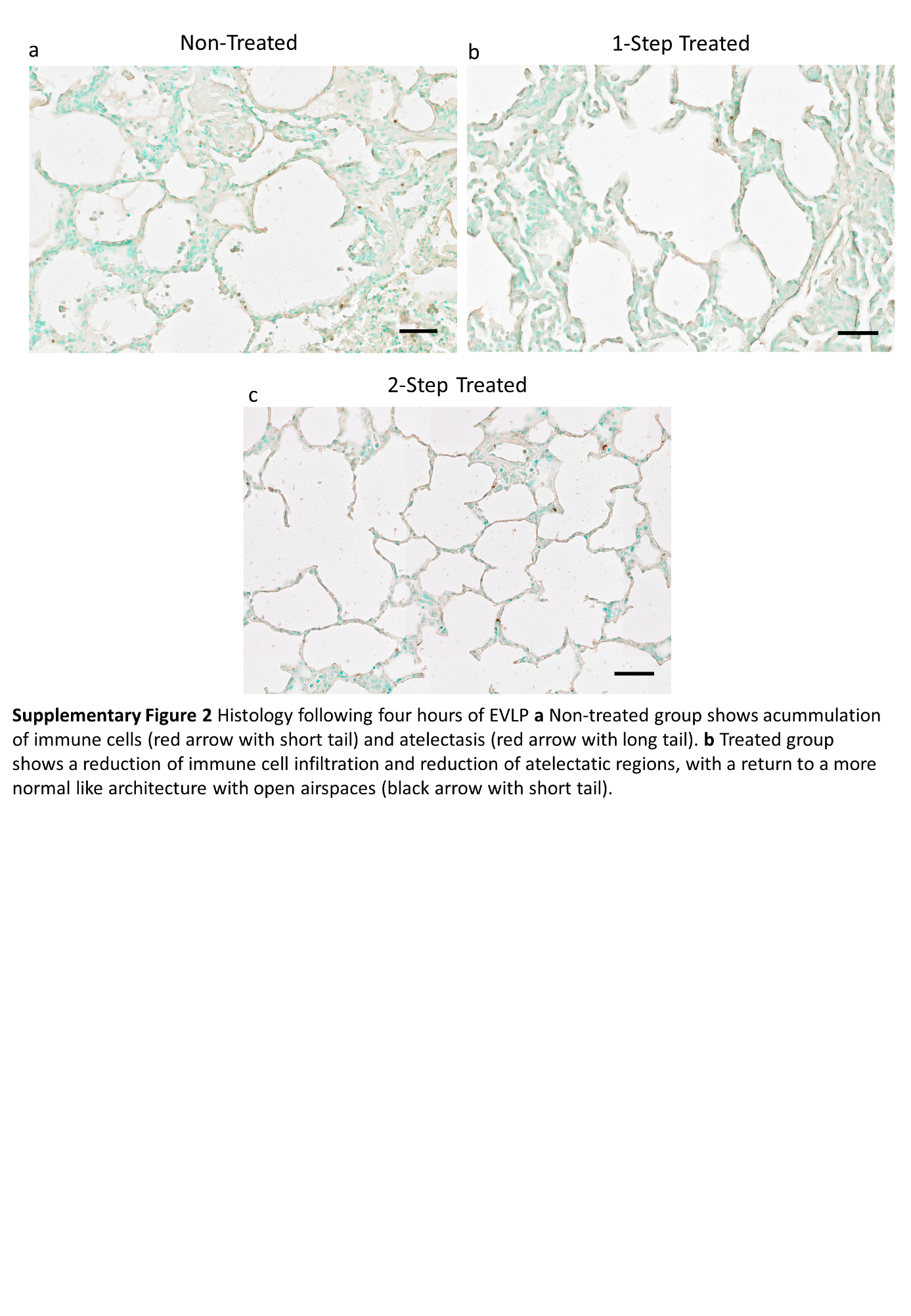
**

**Supplementary Figure 13. Evaluation of late apoptosis in lung tissue following the end of observation in recipients (LTx end) representative of n=16 lungs.** Bright field images of terminal deoxynucleotidyl transferase dUTP nick end labeling (TUNEL) staining (brown) as marker for late apoptosis with methyl green as counter stain with representative images of non-treated lungs in **a** one-step treatment in **b** and two-step treated lungs in **c.**  Scale bars represent 50 µm.

**
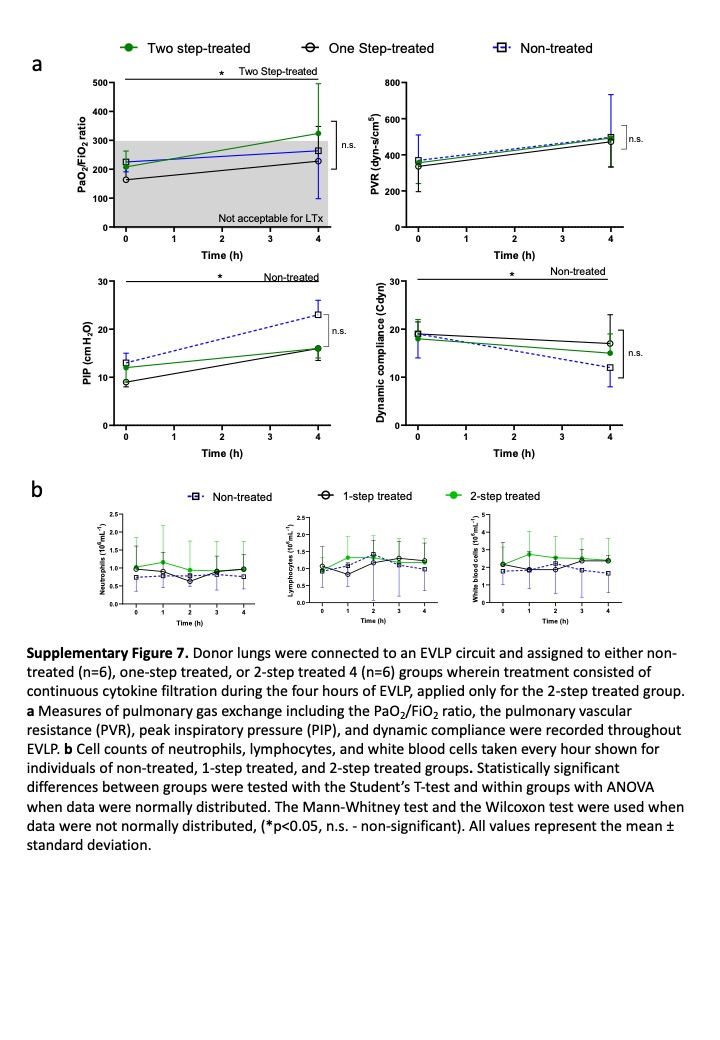
**

**Supplementary Figure 14. Pulmonary gas exchange and peripheral differential blood cell counts during ex vivo lung perfusion (EVLP).** Donor lungs were connected to an EVLP circuit and assigned to either non-treated (n=6), one-step treated (n=4), or 2-step treated 4 (n=6) groups wherein treatment consisted of continuous cytokine filtration during the four hours of EVLP, applied only for the 2-step treated group. **a** Measures of pulmonary gas exchange including the PaO_2_/FiO_2_ ratio, the pulmonary vascular resistance (PVR), peak inspiratory pressure (PIP), and dynamic compliance were recorded throughout EVLP. **b** Cell counts of neutrophils, lymphocytes, and white blood cells taken every hour shown for individuals of non-treated, 1-step treated, and 2-step treated groups. Statistically significant differences between groups were tested with the two-sided Student’s T-test and within groups with ANOVA when data were normally distributed. The two-sided Mann-Whitney test and the Wilcoxon test were used when data were not normally distributed, (*p<0.05, n.s., non-significant). All values represent the mean ± standard deviation.

**
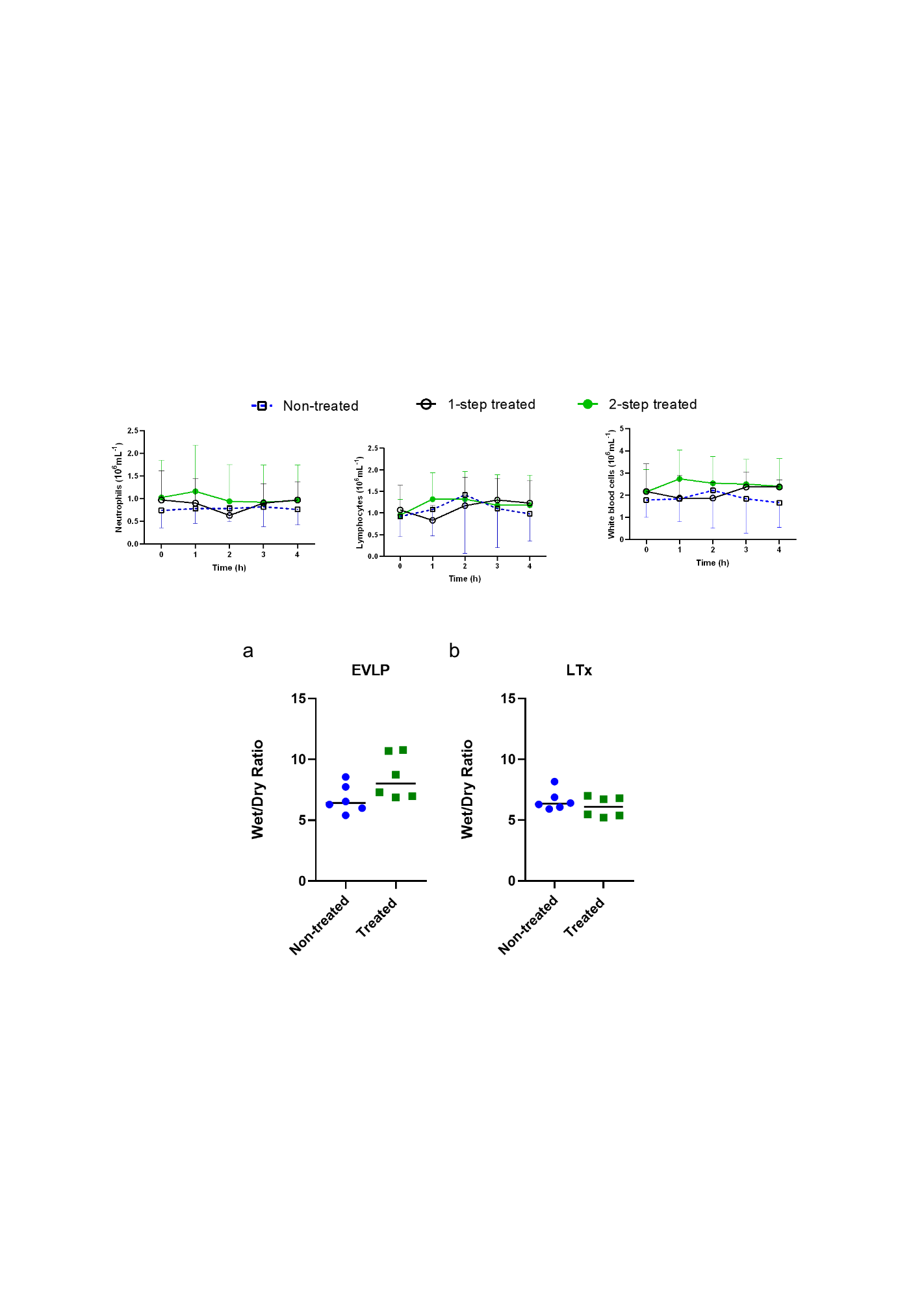
**

**Supplementary Figure 15. Wet/dry ratios of lung biopsy tissue.** **a** Biopsies obtained at the end of ex vivo lung perfusion (EVLP). **b** Biopsies obtained at the end of lung transplantation (LTx). All graphs represent data from either the two-step treated donor lungs (n=6, green squares) or non-treated lungs (n=6, blue circles). Statistically significant differences between groups were tested with the two-sided Mann Whitney test. All values represent the mean ± standard deviation.

**Supplementary Videos**

**Supplementary video 1.** Setup of cytokine adsorption system using extracorporeal hemoperfusion in the post-transplantation recipient.

**Supplementary Tables**

**Supplementary Table 1. Physiological status of donors treated with lipopolysaccharide (LPS) over time until confirmation of acute respiratory distress syndrome (ARDS).** The values for the treated donors are shown in the gray rows and the non-treated donors in the white rows. Vitals: oxygen saturation (Sat), heart rate (HR), systolic blood pressure (SBP), diastolic blood pressure (DBP), mean arterial pressure (MAP), central venous pressure (CVP), temperature (Temp); hemodynamic variables: systolic pulmonary pressure (SPP), diastolic pulmonary pressure (DPP), mean pulmonary pressure (MPP), pulmonary artery wedge pressure (Wedge), cardiac output (CO), systemic vascular resistance (SVR). Blood gas parameters: pH, partial pressure of oxygen (PaO_2_), partial pressure of carbon dioxide (PaCO_2_), hemoglobin (Hb), lactate, base excess (BE), partial pressure of oxygen divided by fraction of inspired oxygen (PaO_2_/FiO_2_). Mechanical ventilator settings with volume-controlled ventilation: minute volume (MV), peak inspiratory pressure (PIP), peak inspiratory pressure, positive end-expiratory pressure (PEEP), tidal volume (Vt), dynamic compliance (Cdyn), respiratory rate (RR), fraction of inspired oxygen (FiO_2_). The values for the 2-step treated animals are shown in the light gray rows and 1-step treated animals in the dark gray rows with bold text while the non-treated animals shown in the white rows.

|  | Baseline | 30 min | 60 min | 90 min | 120 min | Confirmed ARDS |
| --- | --- | --- | --- | --- | --- | --- |
| *Sat (%)* | 99±1.5 | 97±2.6 | 95±3.6 | 97±1.7 | 97 ±2.8 | 92±9.4 |
|  | **100±0.0** | **100±0.0** | **100±0.0** | **100±0.0** | **100±0.0** | **100±0.0** |
|  | 98.3±1.5 | 96.5±2.7 | 96.8±3.1 | 97.6±2.7 | 96.8±3.2 | 95±2.3 |
| *HR (bpm)* | 64.0±6.6 | 92.0±22.4 | 119±10.9 | 124±16.7 | 117±28.3 | 119±1.4 |
|  | **93.5±24.5** | **134±13.1** | **130±10.1** | **140±15.1** | **134±14.1** | **147.5±19.5** |
|  | 70.8±11.9 | 70±8.2 | 114.3±34.1 | 125±13.4 | 131.7±19.8 | 133.8±14.6 |
| *SBP (mmHg)* | 103±13.1 | 109±25.4 | 97.0±7.8 | 99.0±9.9 | 100.0±15.6 | 92.0±0.5 |
|  | **103.5±9.6** | **104.2±14.2** | **103.6±30.6** | **98.6±10.6** | **103.6±30.6** | **102.0±31.0** |
|  | 98.5±8.1 | 116.3±17.5 | 105.3±13.4 | 98.5±6.2 | 108.6±31.1 | 113.8±26.9 |
| *DBP (mmHg)* | 73.0±14.5 | 74.0±23.1 | 64.0±7.0 | 45.0±7.0 | 42±16.3 | 70.0±29.0 |
|  | **70.5±11.9** | **85.5±16.0** | **84.3±43.0** | **85.3±33** | **84.6±40** | **85.0±37.3** |
|  | 68.8±6.2 | 79.5±19.6 | 69.5±18.3 | 49.4±13.0 | 56.0±13.9 | 56.3±13.9 |
| *MAP (mmHg)* | 85.0±15.7 | 87.0±23.4 | 74±8.6 | 62±7.9 | 58.0±21.2 | 65.3±12.1 |
|  | **83.7±11.2** | **93.7±16.6** | **91.6±38.5** | **90.8±30.5** | **95.6±34.5** | **88.3±39.2** |
|  | 81.2±6.0 | 96.8±18.7 | 82.3±15.4 | 63.8±10.8 | 70.0±14.2 | 69.5±13.6 |
| *CVP (mmHg)* | 5.0±2.2 | 9.0±6.1 | 5.0±3.2 | 4.0±2.5 | 6.0±2.1 | 6.1±2.0 |
|  | **8.2±3.5** | **10.3±2.6** | **10.8±3.8** | **10.0±3.8** | **10.5±2.7** | **8.0±3.2** |
|  | 8.0±2.1 | 8.3±3.5 | 7.3±3.9 | 5.6±2.5 | 6.7±3.8 | 6.2±2.6 |
| *Temp (°C)* | 38.0±1.1 | 38.0±1.4 | 38.0±1.7 | 37.0±1.9 | 37.0±1.7 | 37.8±1.4 |
|  | **40.9±0.5** | **41.3±0.6** | **41.3±0.7** | **42.5±0.8** | **43.3±0.9** | **41.5±0.7** |
|  | 37.8±0.9 | 38.2±1.5 | 38.4±1.6 | 38.1±1.6 | 38.1±1.6 | 38.5±1.5 |
| *SPP (mmHg)* | 27.3±6.6 | 68±14.1 | 38.3±11.89 | 36.3±3.5 | 37.5±2.1 | *41.3±11* |
|  | **24.2±4.3** | **45±15.3** | **35.0±8.0** | **32.0±7.0** | **37.0±9.0** | **35.0±6.0** |
|  | 23.8±2.0 | 64.2±31 | 42.3±18 | 38.7±10 | 39±10 | 42.0±9.0 |
| *DPP (mmHg)* | 14.3±3.7 | 37.8±8.9 | 24±6.12 | 20.3±1.5 | 23.5±2.1 | 41.5±38.3 |
|  | **14.2±6.8** | **31.5±10.4** | **26.0±14.7** | **27.0±13.6** | **28.0±13.5** | **26.0±12.2** |
|  | 12.2±5 | 31.0±12.0 | 25.8±8.0 | 22.8±8.0 | 25.8±5.0 | 26.0±8.0 |
| *MPP (mmHg)* | 20.1±5 | 47.1±10.8 | 29.8±7.6 | 27.6±1.1 | 31.0±1.4 | 32.1±7.4 |
|  | **19±4.0** | **36±12.3** | **31.4±8** | **30.7±10** | **32.3±8** | **30.0±8.4** |
|  | 18.0±3 | 39.0±15.0 | 33.0±12 | 30.0±7.0 | 32.0.0±6 | 32.0±6.0 |
| *Wedge (mmHg)* | 8.3±2.8 | 11.8±4.2 | 8.1±1.9 | 6.3±1.2 | 7.5±2.1 | 8.5±1.7 |
|  | **12.7±4.5** | **8.5±1.9** | **9.2±1.6** | **9.3±1.5** | **9.2±1.3** | **9.0±1.4** |
|  | 11.5±2 | 16.5±9 | 13.5±9 | 12.7±3 | 10.0±3 | 10±3.0 |
| *CO (L/min)* | 4.2±1.1 | 4.6±1.7 | 5.8±2.5 | 7.5±3.1 | 6.6±4.3 | 5.9±2.8 |
|  | **3.2±0.6** | **3.9±1.08** | **4.8±1.2** | **5.9±2.8** | **5.7±1.4** | **5.7±2.0** |
|  | 4.1±1.0 | 4.7±1.0 | 5.5±2.0 | 6.1±2.0 | 5.4±1.0 | 5.0±5.0 |
| *SVR (DS/cm^5^)* | 1485.8±380.0 | 1563.6±795.4 | 1129.1±349.1 | 652.6±220.9 | 693.5±153.4 | 999.5±428.6 |
|  | **1669.0±206.9** | **1767.5±717.0** | **1172.5±477.0** | **1186.5±450** | **1168.5±467.0** | **1181.5±497.0** |
|  | 1442±324.0 | 1500±239.0 | 1216±443.0 | 881.0±122.0 | 1023.0±401.0 | 998.0±376.0 |
| *PVR (DS/cm^5^)* | 215.3±85.5 | 597.5±352.3 | 359±231.3 | 257.66±137.5 | 340.0±131.5 | 406±259.4 |
|  | **164.0±63.1** | **219.2±36.4** | **337±192.4** | **367±152.4** | **328.0±182.4** | **327.3±172.4** |
|  | 120.5±39.0 | 435±239.0 | 375.6±205 | 331.8±91 | 310.2±117.0 | 347.0±110.0 |
| *CI (L/min/m^2^)* | 2.7±0.6 | 3.1±0.8 | 3.8±1.4 | 4.7±1.6 | 4.3±2.5 | 3.8±1.6 |
|  | **3.1±0.6** | **3.8±1.0** | **5.6±2.2** | **6.1±2.1** | **5.8±3.2** | **5.4±2.2** |
|  | 2.7±0.0 | 3.3±1.0 | 3.7±1.0 | 3.5±1.0 | 3.5±0.0 | 4.0±1.0 |
| *pH* | 7.45±0.1 | 7.3±0.1 | 7.2±0.1 | 7.3±0.0 | 7.3±0.0 | 7.2±0.05 |
|  | **7.4±0.0** | **7.3±0.05** | **7.5±0.08** | **7.8±0.07** | **7.7±0.08** | **7.3±0.1** |
|  | 7.4±0.1 | 7.4±0.1 | 7.3±0.1 | 7.3±0.1 | 7.2±0.1 | 7.3±0.1 |
| *Hb (g/L)* | 92±9.3 | 102.4±10.6 | 106±10.5 | 106±10.4 | 103.0±14.1 | 103. 3±9.9 |
|  | **83.0±2.1** | **87.0±4.3** | **82.5±5.3** | **81.8±6.1** | **80.5±5.0** | **77.0±7.0** |
|  | 96±12.3 | 103.2±8.1 | 106±3.8 | 103±3.6 | 96.3±2.1 | 102.7±6.0 |
| *Lactate (mmol/L)* | 1.5±0.2 | 1.4±0.5 | 1.7±0.3 | 2.1±0.3 | 2.6±0.3 | 2.2±0.5 |
|  | **1.7±0.7** | **2.7±1.2** | **2.9±1.3** | **2.8±1.6** | **2.3±1.9** | **3.1±1.3** |
|  | 1.6±0.7 | 1.2±0.4 | 1.4±0.4 | 2.2±0.7 | 2.9±1.0 | 2.2±1.0 |
| *BE (mmol/L)* | 5.3±3.3 | 3.8±2.5 | 2.8±2.1 | 2.7±1.7 | 0.9±0.4 | 1.62±1.7 |
|  | **4.7±2.5** | **3.3±2.4** | **2.08±1.8** | **2.26±1.9** | **3.07±1.2** | **2.8±1.8** |
|  | 3.5±2.1 | 3.5±1.4 | 2.5±1.1 | 1.7±1.4 | 2.7±0.3 | 2.0±1.5 |
| *PaO_2_/FiO_2_ (mmHg)* | 519.9±26.9 | 346±169.5 | 301.1±135.7 | 282.1±138.7 | 281.9±103.6 | 208.2±55.5 |
|  | **415.1±14.1** | **333.3±101.5** | **211.5±25.1** | **204.7±43.4** | **204.7±43.4** | **204.8±33.6** |
|  | 514.2±30.1 | 373±155.3 | 367.6±126.4 | 315.9±110.4 | 294.5±124.8 | 225.3±33.6 |
| *MV (L/min)* | 7.6±1.3 | 7.6±1.3 | 7.75±1.7 | 8±1.7 | 7.5±2.1 | 8.1±1.5 |
|  | **7.5±1.0** | **7.5±0.7** | **7.9±0.8** | **7.9±0.8** | **7.9±0.8** | **7.7±0.9** |
|  | 8.3±1.1 | 8.5±1.2 | 8.9±1.7 | 9±1.9 | 7.3±1.1 | 9.5±1.8 |
| *Max. Pressure (cmH_2_O)* | 16.4±2.8 | 18.5±2.9 | 20.1±2.9 | 19±1.7 | 21±1.4 | 21.1±2.6 |
|  | **16.7±0.9** | **19.2±1.5** | **18.3±1.5** | **18.3±1.5** | **18.3±1.5** | **18.0±2.4** |
|  | 16.9±3.4 | 19.5±3.4 | 20.3±5.4 | 20±5.1 | 18±1.2 | 21.3±4.9 |
| *PEEP (cmH_2_O)* | 5±0.0 | 5±0.0 | 5±0.0 | 5±0.0 | 5±0.0 | 5.1±0.0 |
|  | **5±0.0** | **5±0.0** | **5±0.0** | **5±0.0** | **5±0.0** | **5±0.0** |
|  | 5±0.0 | 5±0.0 | 5±0.0 | 5±0.0 | 5±0.0 | 5±0.0 |
| *Vt (mL)* | 387.7±48.2 | 394.8±40.4 | 388.0±36.5 | 386.0±35.5 | 376.5±40.3 | 386.3±37.8 |
|  | **404.2±44.4** | **409.5±21.0** | **415.2±21.3** | **415.2±21.3** | **415.2±21.3** | **406.0±16.1** |
|  | 390.3±54.3 | 375.2±36.8 | 378.7±40.1 | 377.8±20.6 | 365.3±9.2 | 389.3±33.7 |
| *Cdyn (mL/cmH_2_O)* | 35.4±10.2 | 31.0±8.5 | 26.5±5.5 | 27.9±5.5 | 23.5±0.4 | 24.7±5.6 |
|  | **24.7±3.7** | **20.7±2.4** | **16.7±11.3** | **16.7±11.3** | **16.7±11.3** | **20.5±2.0** |
|  | 35.7±14.4 | 27.0±4.8 | 26.3±5.7 | 26.7±5.8 | 27.5±2.2 | 25.1±5.2 |
| *RR (breaths/min)* | 19.3±0.8 | 19.3±0.8 | 22±2.7 | 21.6±2.5 | 22.0±4.9 | 21.6±3.1 |
|  | **24.7±3.7** | **20.7±2.4** | **16.7±11.3** | **16.7±11.3** | **16.7±11.3** | **26.5±1.7** |
|  | 20.2±2.1 | 22.3±3.6 | 23.0±3.6 | 23.2±4.0 | 23.2±2.8 | 23.7±3.5 |

*Two-step treated: Light grey; One-step treated: Dark grey, bold text; Non-treated: White*

**Supplementary Table 2.** **Physiological status of recipients with or without treatment post right pneumonectomy.** The values for the treated recipients are shown in the gray rows and the non-treated in the white rows. Vitals: oxygen saturation (Sat), heart rate (HR), systolic blood pressure (SBP), diastolic blood pressure (DBP), mean arterial pressure (MAP), central venous pressure (CVP), temperature (Temp). Hemodynamic variables: systolic pulmonary pressure (SPP), diastolic pulmonary pressure (DPP), mean pulmonary pressure (MPP), cardiac output (CO), systemic vascular resistance (SVR), pulmonary vascular resistance (PVR). Blood gas parameters: hemoglobin (Hb), lactate, base excess (BE). Mechanical ventilator settings with volume-controlled ventilation: minute volume (MV), peak inspiratory pressure (PIP), peak inspiratory pressure, positive end-expiratory pressure (PEEP), tidal volume (Vt), respiratory rate (RR). The values for the 2-step treated animals are shown in the light gray rows and 1-step treated animals in the dark gray rows with bold text while the non-treated animals shown in the white rows.

|  | Baseline | 15min | 30min | 1h | 1.5h | 2h | 2.5h | 3h | 3.5h | 4h |
| --- | --- | --- | --- | --- | --- | --- | --- | --- | --- | --- |
| *Sat (%)* | 96.1±3.3 | 95.5±3.2 | 95.5±4.2 | 94.5±3.3 | 95±4 | 94±4.9 | 94.1±4.1 | 93.6±5 | 94±7.2 | 96±2.5 |
|  | **100±0** | **100±0** | **100±0** | **100±0** | **100±0** | **100±0** | **100±0** | **100±0** | **100±0** | **100±0** |
|  | 96.8±1.5 | 96.6±1.8 | 95.6±2.2 | 96±4.1 | 95±4.1 | 94.8±4.1 | 94±3.6 | 95±4.0 | 95±3.8 | 95.2±4.3 |
| *HR (bpm)* | 91±17.2 | 93.7±25.2 | 88.8±21.6 | 90.7±29.7 | 97.5±39.1 | 93±29.8 | 93.8±28.9 | 92±33.7 | 88.5±24.6 | 83.3±19.0 |
|  | **107±10.5** | **117.5±15.9** | **121.7±12.9** | **124.5±14.4** | **122.5±14.3** | **126.75±16.3** | **132.5±15** | **133±14.6** | **132.2±11.5** | **133.3±12.5** |
|  | 79±17.0 | 77.8±18.7 | 85.6±13.0 | 92.8±18.3 | 94±24.1 | 88±24.0 | 90.3±33.2 | 87±28.1 | 89.4±38.8 | 89.8±34.2 |
| *SBP (mmHg)* | 106.6±13.1 | 100.8±5.6 | 105.2±12.9 | 100±6.7 | 102.4±7.0 | 105.4±8.9 | 104±8.8 | 96±14.9 | 100±5.9 | 101.6±7.5 |
|  | **102±8.2** | **102±16.1** | **104.2±23.5** | **105.5±21.2** | **106.7±23.6** | **99.7±11.8** | **97±12.9** | **102.2±9.2** | **100±9.8** | **100±9.8** |
|  | 109.4±8.5 | 106.4±13.5 | 101.8±8.0 | 109±6.8 | 111.2±8.3 | 110±6.1 | 102.4±12.8 | 98±16.7 | 101±21.1 | 105±12.5 |
| *DBP (mmHg)* | 63±19.0 | 56±3.7 | 66.2±16.9 | 55±5.4 | 53.6±4.2 | 53±13.2 | 55±7.5 | 51±14.7 | 49.4±16.2 | 47.4±17.5 |
|  | **61.2±9.5** | **59.2±13.4** | **61.7±20.9** | **61.7±19.5** | **64.5±22.6** | **55.7±9.5** | **53.7±12.8** | **54.5±10.6** | **54.2±9.4** | **55.2±8.4** |
|  | 72±8.6 | 74±11.4 | 64.4±2.6 | 68±12.3 | 68.2±9.1 | 70±11.0 | 65±5.5 | 58±16.1 | 59.2±17.6 | 65.6±3.1 |
| *MAP (mmHg)* | 80.2±20.1 | 73.8±6.8 | 80.4±14.2 | 72±5.2 | 72±4.8 | 70±15.1 | 69±15.3 | 60.6±12.9 | 69±19.9 | 64.8±21.1 |
|  | **73.7±12.6** | **75.2±17.2** | **78.2±24.5** | **78.5±22.4** | **80.7±22.6** | **72.5±11** | **69.2±14.6** | **71.2±11.7** | **72.5±10.6** | **71.5±11.6** |
|  | 87.8±11.4 | 88.2±12.4 | 79.6±4.9 | 83±12.4 | 84±9.9 | 85±11.4 | 81±6.7 | 72.8±18.5 | 74±19.0 | 80.4±6.1 |
| *CVP (mmHg)* | 6.8±2.3 | 6.4±1.5 | 7±1.4 | 7±2.3 | 6.2±1.1 | 6.4±1.5 | 6.4±1.9 | 7±2.2 | 7±2.3 | 7±2.5 |
|  | **8.7±2.8** | **10.2±2.6** | **11±2.5** | **10.5±2.3** | **11.2±3.3** | **10.5±3** | **11±3.4** | **11±2.9** | **10.7±2.6** | **10.2±2.8** |
|  | 7.6±3.4 | 6.8±2.2 | 7±2.4 | 7±3.1 | 6.4±2.7 | 6.4±2.7 | 6.0±2.7 | 6.0±2.7 | 6.0±2.7 | 6.0±3.1 |
| *Temp (°C)* | 38.6±0.6 | 38.4±0.9 | 38.3±0.9 | 38.2±0.9 | 38.2±0.8 | 38.3±0.8 | 38.2±0.8 | 38.1±0.8 | 38.2±0.7 | 38±0.8 |
|  | **39.4±0.4** | **39.05±0.3** | **38.8±0.3** | **38.6±0.5** | **38.4±0.4** | **38.03±0.9** | **38.01±1.2** | **38.8±0.6** | **38.5±0.7** | **38.2±0.6** |
|  | 39.5±0.4 | 39.4±0.4 | 39.4±0.3 | 39.3±0.3 | 39.2±0.4 | 38.9±0.5 | 38.9±0.6 | 38.9±0.6 | 38.7±0.9 | 38.4±1.2 |
| *CO (L/min)* | 4.3±0.9 | 3.5±0.8 | 4±0.1 | 3.9±0.1 | 4.1±0.4 | 4.1±0.6 | 4.1±0.6 | 3.8±0.7 | 3.9±0.6 | 3.7±0.6 |
|  | **4.75±0.8** | **4.2±0.9** | **4.4±0.7** | **4.3±0.5** | **4.0±0.5** | **4.3±0.6** | **4.4±0.6** | **4.4±0.8** | **4.4±0.7** | **4.2±0.8** |
|  | 4.5±0.1 | 4.7±0.1 | 5±1.2 | 5.3±0.8 | 5.3±1.0 | 5.5±0.8 | 5.5±0.9 | 5.7±0.7 | 5.2±0.9 | 5.3±0.9 |
| *SVR (DS/cm^5^)* | 1327±356 | 1182±269 | 1374±480 | 1190±282 | 1181±147 | 1248±231 | 1260±265 | 1277±363 | 1272±194 | 1415±413 |
|  | **1202±452** | **1202±452** | **1201±354** | **1227±288** | **1344±423** | **1187±166** | **1134±196** | **1089±266** | **1159±200** | **1192±240** |
|  | 1180±200 | 1178±160 | 1009±203 | 848±199 | 1030±233 | 999±198 | 1040±198 | 1030±188 | 1088±200 | 1030±139 |
| *PVR (DS/cm^5^)* | 196±62.0 | 273.0±70.0 | 281.0±65.0 | 273±80.0 | 267±101.0 | 244±65.0 | 261±60.0 | 265±88.0 | 276±66.0 | 244±70.0 |
|  | **159.0±152.7** | **227.2±206.2** | **198.5±142.1** | **176±105.6** | **189.5±96.9** | **176±94.9** | **176.5±74.1** | **187.5±71.5** | **177±84.2** | **178±80.2** |
|  | 238.0±60.0 | 253.0±55.0 | 261.0±80.0 | 312±121 | 427±130 | 434±133.0 | 440±123.0 | 429±100.0 | 456±110.0 | 427±98.0 |
| *pH* | 7.4±0.1 | 7.4±0.1 | 7.3±0.1 | 7.4±0.1 | 7.3±0.1 | 7.3±0.2 | 7.3±0.1 | 7.3±0.1 | 7.3±0.1 | 7.3±0.1 |
|  | **7.4±0.05** | **7.4±0** | **7.4±0.04** | **7.3±0.05** | **7.4±0** | **7.3±0.05** | **7.4±1.08** | **7.4±0** | **7.4±1.08** | **7.4±1.02** |
|  | 7.4±0.1 | 7.3±0.1 | 7.3±0.1 | 7.3±0 | 7.3±0.1 | 7.3±0.2 | 7.3±0.2 | 7.3±0.2 | 7.3±0.2 | 7.3±0.2 |
| *Hb (g/L)* | 72±10.2 | 72.2±8.9 | 70.8±10.3 | 70.6±10.0 | 71.6±8.8 | 71.6±8.8 | 70±10.7 | 68.8±11.1 | 68.8±11.1 | 69.4±10.5 |
|  | **51.5±4.2** | **50.3±0.5** | **55±6** | **51±3.9** | **50±2.8** | **49.5±1** | **51±1.7** | **49.2±2.9** | **51.6±3** | **51.8±2** |
|  | 83±3.4 | 85±5.3 | 83.8±6.1 | 85.6±5.7 | 85.4±5.9 | 85.4±5.9 | 85±7.8 | 82±14.4 | 81.6±14.4 | 82.6±10.1 |
| *BE (mmol/L)* | 6.4±3.8 | 5.3±2.8 | 5.5±2.9 | 6.0±2.8 | 5.9±2.4 | 6.8±2.4 | 6.8±3.1 | 7.0±2.5 | 7.5±2.5 | 8.7±2.7 |
|  | **6.6±2.8** | **4.6±3.1** | **4±2.5** | **4.2±1.6** | **3.4±2.4** | **4.2±1.4** | **4.2±2.1** | **3.9±2** | **4.3±2.2** | **4.4±1.2** |
|  | 6.9±4.1 | 5.7±3.3 | 5.9±3.3 | 6.5±3.0 | 7.0±2.7 | 7.0±2.7 | 7.6±3.4 | 8.1±1.8 | 8.1±0.8 | 8.4±1.8 |
| *MV (L/min)* | 9.7±1.8 | 9.9±1.6 | 10.4±2.3 | 10.4±2.3 | 10.4±2.3 | 11.1±2.4 | 10.8±2.1 | 11.1±2.4 | 11.5±2.8 | 11.8±3.0 |
|  | **7.3±1.2** | **7.6±1.2** | **7.3±0.9** | **7.2±0.8** | **7.5±0.7** | **7.3±0.9** | **7.7±1** | **7.7±1** | **7.9±0.7** | **7.8±0.8** |
|  | 10.1±1.5 | 10.4±1.5 | 10.5±1.5 | 10.9±1.4 | 10.8±1.7 | 10.8±1.7 | 11.2±1.2 | 11.7±1.6 | 11.7±1.6 | 11.7±1.6 |
| *Max. Pressure (cmH_2_O)* | 22.0±2.5 | 23.6±2.8 | 23.2±2.7 | 23.2±2.7 | 24.2±3.4 | 23.6±2.9 | 23.8±2.9 | 24.0±3.0 | 23.6±3.0 | 24.0±3.0 |
|  | **26.7±2.2** | **26.2±1.7** | **26.2±1.7** | **26.5±1.9** | **27.0±2.0** | **26.5±1.7** | **27.7±4.1** | **27.5±1.7** | **28.2±1.7** | **27.2±1.8** |
|  | 22.4±3.4 | 25.4±5.4 | 24.6±4.7 | 24.4±4.0 | 24.6±3.7 | 24.6±3.7 | 25.4±3.0 | 25.2±2.6 | 25.2±2.6 | 25.4±2.9 |
| *PEEP (cmH_2_O)* | 6.4±2.2 | 6.6±2.3 | 6.2±2.2 | 6.2±2.2 | 6.0±2.2 | 6.0±2.2 | 6.0±2.2 | 6.0±2.2 | 6.0±2.2 | 6.0±2.2 |
|  | **8.0±0.8** | **7.5±0.5** | **7.5±0.5** | **8.2±1.2** | **8.2±1.2** | **8.2±1.2** | **8.5±1.2** | **8.2±1.2** | **8.5±1** | **8.6±1** |
|  | 6.2±1.6 | 5.6±1.3 | 5.6±1.3 | 5.6±1.3 | 5.6±1.3 | 5.6±1.3 | 5.6±1.3 | 5.6±1.3 | 5.6±1.3 | 5.6±1.3 |
| *Vt (mL)* | 407±58 | 409±53 | 409±54 | 409±54 | 409±54 | 409±54 | 398±38 | 397±38 | 397±38 | 396±35 |
|  | **416±37** | **385±62** | **385±62** | **388±61** | **390±58** | **388±61** | **388±61** | **411±66** | **409±70** | **417±56** |
|  | 429±52 | 399±62 | 405±58 | 404±57 | 401±58 | 401±58 | 386±12 | 381±15 | 381±15 | 385±22 |
| *Cdyn (mL/cmH_2_O)* | 26.4±1.8 | 24.2±3.0 | 24.2±3.4 | 22.7±3.4 | 22.8±2.7 | 22.6±2.0 | 22.3±2.4 | 22.8±2.5 | 22.7±2.4 | 22.7±2.4 |
|  | **26.0±5.8** | **23.7 ±5.1** | **23.7±5.1** | **23.9±5.0** | **25.0±6.1** | **24.0±5.5** | **24.7±5.7** | **25.1±6.3** | **24.5±4.8** | **24.4±4.8** |
|  | 27.1±5.0 | 22.1±5.6 | 22.1±5.2 | 21.6±5.0 | 19.8±2.6 | 19.6±2.0 | 19.7±2.5 | 19.7±2.0 | 20.4±2.5 | 20.4±3.4 |
| *RR (breaths/min)* | 26.4±4.1 | 24.2±3.7 | 24.2±3.1 | 24.2±3.1 | 22.7±3.1 | 22.8±3.1 | 22.6±3.1 | 22.3±3.1 | 22.8±3.3 | 22.7±3.3 |
|  | **26±1.4** | **28.2±4.5** | **28.2±4.5** | **28.7±4.1** | **29.5±4** | **29.5±4** | **29.5±4** | **29.5±4** | **29.5±4** | **29.5±4** |
|  | 23.8±4.0 | 26.4±3.4 | 26.4±3.4 | 27.4±3.8 | 28.8±4.0 | 28.8±4.0 | 29.8±4.9 | 29.8±4.9 | 29.8±4.9 | 29.8±4.9 |
| *SPP (mmHg)* | 27±6 | 41±6 | 38.3±10 | 27±11 | 27±9 | 55±13 | 27±10 | 27±6 | 36±5 | 38±3 |
|  | **30±6** | **32±12.6** | **33±10.6** | **32±9.3** | **32±10.0** | **34.7±9.6** | **33.5±7.7** | **36.2±6.2** | **35±6.6** | **36±6.7** |
|  | 26±4 | 58±18 | 27±5 | 29±5 | 29±4.3 | 33±12 | 37±19 | 39±5.3 | 38±11 | 38±8 |
| *DPP (mmHg)* | 18±11 | 28±6 | 22.8±17 | 21±2 | 23±3.0 | 25±15 | 20±6 | 22±8 | 28.3±3 | 23.5±3 |
|  | **16±2.9** | **17±9.5** | **19±7.4** | **17.2±7.4** | **16.7±6.3** | **17.2±6.2** | **17±6.3** | **16.5±5.2** | **16±4.6** | **17±4.8** |
|  | 15±6.0 | 31±12 | 21±11 | 23.8±5.0 | 21±13 | 23±14.0 | 28±6.6 | 27±18.0 | 20.7±3.0 | 27.4±12.0 |
| *MPP (mmHg)* | 24±2.0 | 57±8 | 28±12 | 23.7±2 | 24±2.2 | 42±10 | 22±12 | 20±8 | 30±6.2 | 31±2 |
|  | **20.7±4.3** | **23.2±10.5** | **24±9.4** | **23.7±6.9** | **23.2±7.2** | **21.2±6.7** | **22.2±6.3** | **23.5±5.8** | **22.5±5.8** | **22.6±5.9** |
|  | 24.0±2.9 | 47±13 | 20±12 | 25.5±9.2 | 24±8.9 | 30±6.8 | 32±15 | 28±9.1 | 39±8.0 | 30±8.2 |

*Two-step treated: Light grey; One-step treated: Dark grey, bold text; Non-treated: White*

**Supplementary Table 3. Overview of exact p-values present in figure 2-5.**

| **Figure** |  | **p-value** |
| --- | --- | --- |
| Figure 2a | TNF-α 60 min after LPS | 0.0005 |
|  | TNF-α 120 min after LPS | 0.0039 |
|  | TNF-α confirmed ARDS | 0.0001 |
|  | IL-10 60 min after LPS | 0.0010 |
|  | IL-10 120 min after LPS | 0.0039 |
|  | IL-10 confirmed ARDS | 0.0010 |
|  | IL-1β confirmed ARDS | 0.0080 |
|  | IL-8 120 min after LPS | 0.0156 |
|  | IL-8 confirmed ARDS | 0.002 |
|  | IL-6 120 min after LPS | 0.0039 |
|  | IL-6 confirmed ARDS | 0.003 |
|  | IL-12 confirmed ARDS | 0.007 |
| Figure 2b | TNF- α | 0.0011 |
|  | IL-10 | 0.0007 |
|  | IL-1β | <0.0001 |
|  | IL-8 | 0.0171 |
|  | IL-6 | 0.0003 |
|  | IL-12 | 0.0141 |
| Figure 2c | Neutrophils 30 min after LPS | 0.0183 |
|  | Neutrophils 60 min after LPS | <0.0001 |
|  | Neutrophils confirmed ARDS | <0.0001 |
|  | Lymphocytes 30 min after LPS | 0.0196 |
|  | Lymphocytes 60 min after LPS | <0.0001 |
|  | Lymphocytes confirmed ARDS | <0.0001 |
|  | White blood cells 30 min after LPS | 0.0215 |
|  | White blood cells 60 min after LPS | <0.0001 |
|  | White blood cells confirmed ARDS | <0.0001 |
| Figure 2d | Lung Injury Score | <0.0001 |
|  | TUNEL Score | 0.0317 |
| Figure 3a | PaO_2_/FiO_2_ ratio in treated lungs over EVLP | 0.03 |
| Figure 3d | IL-1β | 0.0216 |
| Figure 3f | Lung Injury Score | 0.0160 |
| Figure 4c | Neutrophils 2h after right pneumonectomy | 0.0500 |
|  | Neutrophils 3h after right pneumonectomy | 0.0417 |
|  | Neutrophils comparison | 0.0277 |
|  | Lymphocytes 1h after right pneumonectomy | 0.004 |
|  | Lymphocytes 3h after right pneumonectomy | 0.0012 |
|  | Lymphocytes 4h after right pneumonectomy | 0.0320 |
|  | Lymphocytes comparison | 0.0002 |
|  | White blood cells 24H | 0.0399 |
|  | White blood cells 36H | 0.0301 |
|  | White blood cells 1h after right pneumonectomy | 0.0353 |
|  | White blood cells 2h after right pneumonectomy | 0.0257 |
|  | White blood cells 3h after right pneumonectomy | 0.0330 |
|  | White blood cells comparison | 0.0285 |
| Figure 4d | Lung Injury Score 1-step vs non-treated | 0.0381 |
|  | Lung Injury Score 2-step vs non-treated | 0.026 |
|  | TUNEL Score 1-step vs non-treated | 0.027 |
|  | TUNEL Score 2-step vs non-treated | 0.006 |
| Figure 5a | PaO_2_/FiO_2_ Ratio 36h | **0.001518** |
|  | PaO_2_/FiO_2_ Ratio 44h | **0.002197** |
|  | PaO_2_/FiO_2_ Ratio 46h | **<0.0001** |
|  | PaO_2_/FiO_2_ Ratio 48h | **<0.0001** |
|  | PaO_2_/FiO_2_ Ratio 0h after right pneumonectomy | 0.001302 |
|  | PaO_2_/FiO_2_ Ratio 1h after right pneumonectomy | 0.000371 |
|  | PaO_2_/FiO_2_ Ratio 1.5h after right pneumonectomy | <0.0001 |
|  | PaO_2_/FiO_2_ Ratio 2h after right pneumonectomy | <0.0001 |
|  | PaO_2_/FiO_2_ Ratio 2.5h after right pneumonectomy | <0.0001 |
|  | PaO_2_/FiO_2_ Ratio 3h after right pneumonectomy | <0.0001 |
|  | PaO_2_/FiO_2_ Ratio 3.5h after right pneumonectomy | <0.0001 |
|  | PaO_2_/FiO_2_ Ratio 4h after right pneumonectomy | <0.0001 |
| Figure 5b | PVR 1-step vs non-treated | 0.019 |
|  | PVR 2-step vs non-treated | 0.026 |
| Figure 5c | PaO_2_/FiO_2_ Ratio 1-step vs non-treated | 0.019 |
|  | PaO_2_/FiO_2_ Ratio 1-step vs 2-step | 0.0095 |
|  | PaO_2_/FiO_2_ Ratio 2-step vs non-treated | 0.0022 |
| Figure 5d | PGD χ^2^ test | 0.006 |
