# Supplementary material for: Reduction of primary graft dysfunction using cytokine adsorption during organ preservation and after lung transplantation
Source: Nat Commun. 2022 Jul 26;13:4173. doi: 10.1038/s41467-022-31811-5 (PMC9325745; doi:10.1038/s41467-022-31811-5)
Supplement: Supplementary file 2 — Reporting Summary [file 41467_2022_31811_MOESM2_ESM.pdf]

nature portfolio

Corresponding author(s): Dr. Sandra Lindstedt

Last updated by author(s): May 14, 2022

Reporting Summary

Nature Portfolio wishes to improve the reproducibility of the work that we publish. This form provides structure for consistency and transparency in reporting. For further information on Nature Portfolio policies, see our Editorial Policies and the Editorial Policy Checklist.

Statistics

For all statistical analyses, confirm that the following items are present in the figure legend, table legend, main text, or Methods section.

n/a	Confirmed	
☐	☒	The exact sample size (n) for each experimental group/condition, given as a discrete number and unit of measurement
☐	☒	A statement on whether measurements were taken from distinct samples or whether the same sample was measured repeatedly
☐	☒	The statistical test(s) used AND whether they are one- or two-sided<br>*Only common tests should be described solely by name; describe more complex techniques in the Methods section.*
☒	☐	A description of all covariates tested
☐	☒	A description of any assumptions or corrections, such as tests of normality and adjustment for multiple comparisons
☐	☒	A full description of the statistical parameters including central tendency (e.g. means) or other basic estimates (e.g. regression coefficient) AND variation (e.g. standard deviation) or associated estimates of uncertainty (e.g. confidence intervals)
☐	☒	For null hypothesis testing, the test statistic (e.g. F, t, r) with confidence intervals, effect sizes, degrees of freedom and P value noted<br>*Give P values as exact values whenever suitable.*
☒	☐	For Bayesian analysis, information on the choice of priors and Markov chain Monte Carlo settings
☒	☐	For hierarchical and complex designs, identification of the appropriate level for tests and full reporting of outcomes
☒	☐	Estimates of effect sizes (e.g. Cohen's d, Pearson's r), indicating how they were calculated

Our web collection on statistics for biologists contains articles on many of the points above.

Software and code

Policy information about availability of computer code

Data collection	GraphPad Prism 9.1, Microsoft Excel Version 16.46
Data analysis	GraphPad Prism 9.1, Fiji ImageJ 1.53M

For manuscripts utilizing custom algorithms or software that are central to the research but not yet described in published literature, software must be made available to editors and reviewers. We strongly encourage code deposition in a community repository (e.g. GitHub). See the Nature Portfolio guidelines for submitting code & software for further information.

Data

Policy information about availability of data

All manuscripts must include a data availability statement. This statement should provide the following information, where applicable:

- Accession codes, unique identifiers, or web links for publicly available datasets
- A description of any restrictions on data availability
- For clinical datasets or third party data, please ensure that the statement adheres to our policy

The authors declare that all data reported in the paper and Supplementary Information will be made available to other investigators if requested. The source data underlying Fig. 2a-d, n; Fig. 3a-f, l; Fig. 4a-d; Fig. 5a-b are provided in the Source Data file.

Field-specific reporting

Please select the one below that is the best fit for your research. If you are not sure, read the appropriate sections before making your selection.

- [x] Life sciences
- [ ] Behavioural & social sciences
- [ ] Ecological, evolutionary & environmental sciences

For a reference copy of the document with all sections, see nature.com/documents/nr-reporting-summary-flat.pdf

Life sciences study design

All studies must disclose on these points even when the disclosure is negative.

Sample size	Power calculations were based on previously published work (Iskender et al, 2017; Iskender et al, 2020) which hten went on to show significant differences between treatment groups.
Data exclusions	As stated in the manuscript, one in the treatment group and one in the non-treated group were excluded in the measurement of BAL and plasma cytokines due to the development of septicemia. This would have an effect on the cytokines not attributable to treatment modality.
Replication	Due to ethical concerns regarding animal use, replication of this study was not conducted as this would require additional sacrifice.
Randomization	Randomization was conducted prior to the start of this study using an excel randomization. After the initial study, a non-randomized third group was added.
Blinding	Within the surgical model, due to the nature of the treatment involving the use of a device, blinding of clinicians providing care to the animals was not possible. Thereafter, analyses were blinded as feasible. This included conducting the multiplex analysis by sending the samples to a facility with assigned code as well as histological processing and scoring by blinded scorers. TUNEL staining and cell counting analysis was also blinded.

Reporting for specific materials, systems and methods

We require information from authors about some types of materials, experimental systems and methods used in many studies. Here, indicate whether each material, system or method listed is relevant to your study. If you are not sure if a list item applies to your research, read the appropriate section before selecting a response.

Materials & experimental systems

n/a	Involved in the study	
x		Antibodies
x		Eukaryotic cell lines
x		Palaeontology and archaeology
	x	Animals and other organisms
x		Human research participants
x		Clinical data
x		Dual use research of concern

Methods

n/a	Involved in the study	
x		ChIP-seq
x		Flow cytometry
x		MRI-based neuroimaging

Animals and other organisms

Policy information about studies involving animals; ARRIVE guidelines recommended for reporting animal research

Laboratory animals	Farm-raised wild-type American Yorkshire pigs (Sus scrofa domesticus), male and female individuals between the ages of 7-9 months.
Wild animals	No wild animals were utilized in this study.
Field-collected samples	No field-collected samples were utilized in this study.
Ethics oversight	The study was approved by the local Ethics Committee for Animal Research (Dnr 5.2.18-4903/16, and Dnr 5.2.18-8927/16) at Lund University

Note that full information on the approval of the study protocol must also be provided in the manuscript.
