# Supplementary material for: Reduction of primary graft dysfunction using cytokine adsorption during organ preservation and after lung transplantation
Source: Nat Commun. 2022 Jul 26;13:4173. doi: 10.1038/s41467-022-31811-5 (PMC9325745; doi:10.1038/s41467-022-31811-5)
Supplement: Supplementary file 3 — Description of Additional Supplementary Files [file 41467_2022_31811_MOESM3_ESM.pdf]

Title: Supplementary Movie 1.

Description: Setup of cytokine adsorption system using extracorporeal hemoperfusion in the post-transplantation recipient.
